# Maternal Gut Inflammation Aggravates Acute Liver Failure Through Facilitating Ferroptosis via Altering Gut Microbial Metabolism in Offspring

**DOI:** 10.1002/advs.202411985

**Published:** 2025-01-14

**Authors:** Caijun Zhao, Lijuan Bao, Ruping Shan, Yihong Zhao, Keyi Wu, Shan Shang, Haiqi Li, Yi Liu, Ke Chen, Naisheng Zhang, Cong Ye, Xiaoyu Hu, Yunhe Fu

**Affiliations:** ^1^ Department of Gynecology China‐Japan Union Hospital of Jilin University Changchun 130033 China; ^2^ Department of Clinical Veterinary Medicine College of Veterinary Medicine Jilin University Changchun 130062 China; ^3^ Department of Neurology China‐Japan Union Hospital of Jilin University Changchun 130033 China; ^4^ Department of Orthopedic Center The First Hospital of Jilin University Changchun 130012 China

**Keywords:** acute liver failure, ferroptosis, gut microbiota, indole‐3‐acetic acid, maternal gut inflammation, offspring

## Abstract

Microbial transmission from mother to infant is important for offspring microbiome formation and health. However, it is unclear whether maternal gut inflammation (MGI) during lactation influences mother‐to‐infant microbial transmission and offspring microbiota and disease susceptibility. In this study, it is found that MGI during lactation altered the gut microbiota of suckling pups by shaping the maternal microbiota in the gut and mammary glands. MGI‐induced changes in the gut microbiota of suckling pups lasted into adulthood, resulting in the exacerbation of acute liver failure (ALF) caused by acetaminophen (APAP) in offspring. Specifically, MGI reduced the abundance of *Lactobacillus reuteri* (*L. reuteri*) and its metabolite indole‐3‐acetic acid (IAA) level in adult offspring. *L. reuteri* and IAA alleviated ALF in mice by promoting intestinal IL‐22 production. Mechanistically, IL‐22 limits APAP‐induced excessive oxidative stress and ferroptosis by activating STAT3. The intestinal abundances of *L. reuteri* and IAA are inversely associated with the progression of patients with ALF. Overall, the study reveals the role of MGI in mother‐to‐infant microbial transmission and disease development in offspring, highlighting potential strategies for intervention in ALF based on the IAA‐IL‐22‐STAT3 axis.

## Graphical abstract

This study demonstrates that MGI alters microbial profiles in both the maternal gut and mammary gland, leading to altered gut microbiota in nursed pups and adult offspring. Specifically, MGI‐induced reductions in *L. reuteri* abundance and IAA levels in adult offspring exacerbate APAP‐induced ALF. IAA mitigates APAP‐induced hepatic ferroptosis through the production of IL‐22 and subsequent activation of the STAT3 pathway.

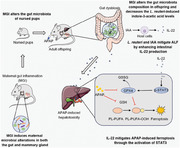

## 1 Introduction

Microbial transmission between mothers and their offspring during the perinatal period endows the offspring with different disease outcomes later in life by altering their microbial, metabolic, and immune profiles.^[^ ^1^, ^2^ ^]^ Although maternal microbes may be vertically transferred from the mother to the fetus,^[^ ^3^, ^4^ ^]^ the delivery mode and breastfeeding status are the predominant factors determining the colonization and development of the newborn microbiota.^[^ ^5^, ^6^, ^7^, ^8^ ^]^ Many factors, such as diet, influence the microbial profile of the mammary gland during the perinatal period and the susceptibility of offspring to diseases.^[^ ^9^, ^10^ ^]^ For example, maternal obesity caused by a high‐fat diet induces cognitive and social dysfunction in the offspring, whereas supplementing with a high‐fiber diet, either in the mother or the offspring, mitigates these changes.^[^ ^11^ ^]^ In contrast, maternal fiber deprivation changed the gut microbiota in offspring, resulting in low‐grade inflammation and a predisposition to obesity.^[^ ^12^ ^]^ Another factor that regulates microbial transmission between mothers and offspring is the maternal status. It has been reported that maternal immune activation, especially that caused by inflammation or infection, changes the gut microbiota of pregnant mice, which causes intestinal inflammation in offspring by altering the chromatin landscape of CD4^+^ T cells.^[^ ^13^ ^]^ Studies have also shown that maternal gut inflammation (MGI) during pregnancy aggravates inflammation in offspring by shaping tissue‐specific immunity.^[^ ^14^ ^]^ However, whether and how MGI during lactation affects the gut microbiota and subsequent disease outcomes in offspring remains unknown.

Microbial tryptophan (Trp) metabolism plays an important role in modulating host‐microbial interaction, in which Trp is metabolized into aryl hydrocarbon receptor (AhR) ligands, including indole‐3‐acetic acid (IAA), 5‐hydroxyindoleacetate (5‐HIAA) and indole propionic acid.^[^ ^15^ ^]^ Endogenous ligand‐mediated AhR activation serves as an effective regulator of host homeostasis in multiple ways, including by promoting interleukin‐22 (IL‐22) production,^[^ ^16^ ^]^ inhibiting the activation of inflammatory signaling pathways^[^ ^17^, ^18^ ^]^ and modulating vascular endothelial cell activation,^[^ ^19^, ^20^ ^]^ leading to an essential role in barrier repair, inflammation, and disease tolerance.^[^ ^21^ ^]^ Among these regulators, AhR‐mediated IL‐22 production by a variety of commensal microbes, including *Lactobacillus*, is widely known to regulate damage repair and barrier function.^[^ ^22^ ^]^ Breastfeeding facilitates the intestinal colonization and expansion of *Bifidobacterium* species, which promotes AhR ligand production and CD4^+^ T‐cell‐mediated immune responses.^[^ ^23^ ^]^ These findings indicate that maternal status may influence the AhR ligand levels in offspring, altering disease outcomes.

Acetaminophen (APAP) overdose‐induced acute liver failure (ALF) is the most common and serious disease in the world, especially in developed countries.^[^ ^24^ ^]^ In general, excessive APAP is metabolized by cytochrome p450 2E1 (CYP2E1) into toxic N‐acetyl‐p‐benzoquinone imine (NAPQI), which binds to functional proteins to form APAP‐protein adducts, resulting in excessive oxidative stress, ferroptosis and ALF.^[^ ^25^ ^]^ Notably, NAPQI can be rapidly detoxified by glutathione (GSH), an antioxidant that reduces reactive oxygen species (ROS) via glutathione peroxidase 4 (GPX4), one of the central regulators of ferroptosis.^[^ ^26^, ^27^ ^]^ Hence, enhancing the GSH/GPX4 axis may serve as a potential strategy for intervention in APAP‐induced ferroptosis and ALF. Interestingly, recent studies have shown that APAP‐induced ALF is closely associated with the gut microbiota.^[^ ^25^, ^28^, ^29^ ^]^ For example, changes in the gut microbiota driven by circadian rhythm regulate ALF susceptibility.^[^ ^28^ ^]^ Another study showed that daidzein (DA) liberated by *Lactobacillus vaginalis* inhibits ALF development by limiting ferroptosis.^[^ ^25^ ^]^ However, whether and how changes in the gut microbiota of offspring regulated by maternal‐to‐infant microbial transmission affect APAP induction in ALF are unknown.

## 2 Results

### 2.1 MGI During Lactation Alters the Gut Microbiota of Nursed Pups

**Figure 1:**
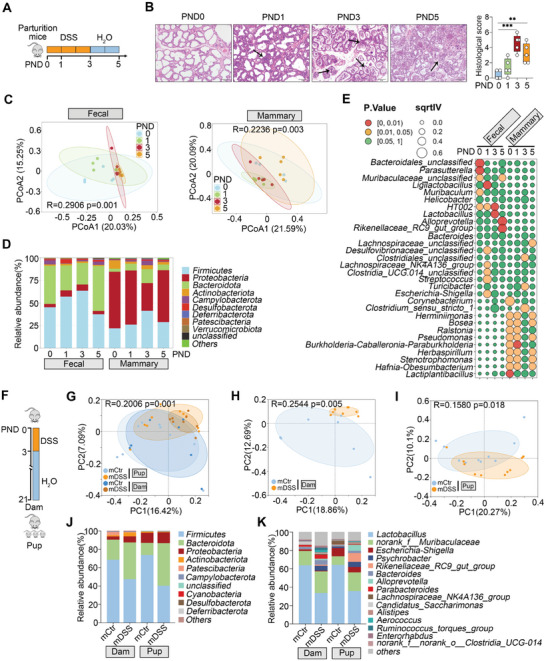
MGI during lactation alters the gut microbiota of nursed pups. A) Schematic diagram of the induction of gut inflammation. Lactating mice were treated with 3% DSS for three continuous days from PND 0 to PND 3, and the treated DSS was replaced with water alone from PND 3 to PND 5. B) Representative H&E‐stained images and histological scores of the mammary glands. Scale bars, 50 µm. C) PCoA score plots of the gut and mammary microbiota of indicated mice. D) Fecal and mammary microbial compositions at the phylum level from indicated mice. E) Taxonomy indicator bubble plot. F) Schematic diagram of the induction of gut inflammation. Lactating mice were treated with 3% DSS for three continuous days from PND 0 to PND 3, followed by free access to drinking water until PND 21. G) PCoA score plots of the gut microbiota in both dams and pups. H, I) Gut microbial structures in different dams (H) and pups (I) were analyzed using PCoA score plots. J, K) Gut microbial compositions at the phylum and genus levels. The data are expressed as box plots (n = 5). ^**^ *p* < 0.01 and ^***^ *p* < 0.001 by one‐way ANOVA followed by Tukey's test (B).

Lactating mice were treated with 3% DSS for three days at postnatal day (PND) 0, followed by water for two days to induce gut inflammation^[^ ^30^ ^]^ (**Figure** **1**A). The mice developed obvious gut inflammation at PND3 and PND5 (Figures S1A,B, Supporting Information). Next, increased mammary inflammatory infiltration was detected in the mice at PND3, and this infiltration was lower at PND5 than at PND3 (Figure 1B). Similarly, the levels of mammary proinflammatory cytokines, including TNF‐α, IL‐1β, and IL‐6, were increased from PND1 after DSS treatment, peaked at PND3, and decreased after DSS removal (PND5) (Figure S1C, Supporting Information), suggesting that gut inflammation can induce mammary inflammatory responses during lactation. Increased liver and systemic inflammation were also observed at PND3 compared with PND0 (Figures S1D–F, Supporting Information). These results were also confirmed by increased serum liposaccharide (LPS) levels during gut inflammation (Figure S1G, Supporting Information).

Next, slight decreases in observed species and alpha diversity were observed in the fecal samples at PND1 and progressed at PND3, but increased after DSS removal (Figure S1H, Supporting Information). Interestingly, the observed species and alpha diversity indices in the mammary glands were the opposite of those of the gut microbiota (Figure S1I, Supporting Information). Significant separations of the gut and mammary microbial structures were observed at different time points after DSS treatment and during the recovery phase (Figure 1C). At the phylum level, increased *Firmicutes* and *Proteobacteria* and decreased *Bacteroidota* abundance were detected in the gut at PND1 and PND3, and these changes were reversed at PND5 compared with PND3 (Figure 1D). Specifically, the *Desulfobacterota* abundance was greater and the *Campylobacterota* abundance was lower at PND1 than at PND0, and these alterations were reversed from PND3 (Figure 1D). Unlike those in the gut, the abundance of mammary *Proteobacteria* was reduced at PND3 and reversed at PND5, whereas the abundances of *Firmicutes*, *Bacteroidota*, and *Campylobacterota* abundances were increased after DSS treatment and reversed after DSS removal (Figure 1D). However, some microbes in the mammary glands, such as *Actinobacteriota*, are lastingly reduced during gut inflammation (Figure 1D). At the genus level, different gut and mammary microbial compositions were observed during the DSS treatment and the recovery phases (Figure S1J, Supporting Information). Some microbes, including *Alloprevotella* in the gut and *Herminiimonas*, *Bosea*, *Ralstonia*, and *Stenotrophomonas* in the mammary gland, were reduced after DSS treatment and reversed after DSS removal, indicating space‐specific bacterial composition regulated by gut inflammation (Figure 1E). In addition, fecal *Lactobacillus* and *HT002* were enriched at PND3 after DSS treatment (Figure 1E). Notably, the changes in *Escherichia_Shigella*, *Clostridium_sensu_stricto_1*, *Streptococcus*, and *Clostridia_UCG.014_unclassified* were consistent in the gut and the mammary gland, with both showing an increase during the DSS treatment group and a decrease during the recovery phase (Figure 1E).

We next investigated whether MGI affects the gut microbial composition of mothers and nursed pups during the weaning period (Figure 1F). No significant differences in alpha diversity indices were detected between the MGI (mDSS) and control (mCtr) groups in dams and pups (Figure S2A, Supporting Information). However, significant separation of the gut microbial structure among the differently treated dams and pups was detected using PCoA (Figures 1G–I). At the phylum level, the relative abundances of *Firmicutes* and *Patescibacteria* were depleted, and those of *Bacteroidota*, *Proteobacteria*, and *Actinobacteriota* were enriched in the guts of the mDSS‐dams compared with those of the mCtr‐dams (Figure 1J; Figure S2B, Supporting Information). Consistent with those in the dams, the relative abundances of *Firmicutes* and *Patescibateria* were also depleted, and *Bacteroidota* was enriched in the guts of mDSS‐pups compared with those of mCtr‐pups, whereas the abundances of *Proteobacteria* and *Actinobacteriota* showed no significant differences (Figure 1J; Figure S2B, Supporting Information). At the genus level, both mDSS dams and pups had depleted *Lactobacillus* and *Escherichia_Shigella* and enriched *Psychrobacter* and *Rikenellaceae_RC_gut_group* abundances compared with those in the control dams and offspring, respectively (Figure 1K). LEfSe confirmed that MGI depleted the relative abundance of *Lactobacillus* in both dams and pups (Figure S2C, Supporting Information). Together, these results indicate that MGI alters the gut microbiota of nursed pups.

### 2.2 MGI Lastingly Impacts Offspring Microbiota and Exacerbates ALF in Offspring

**Figure 2:**
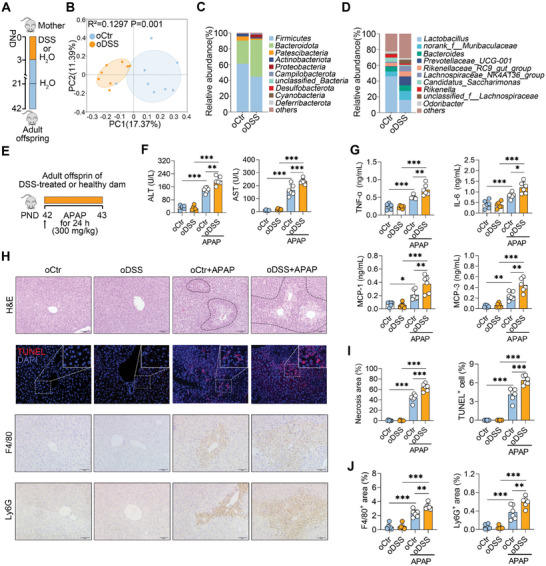
MGI lastingly impacts offspring microbiota and exacerbates ALF in offspring. A) Schematic diagram. Lactating mice were treated with 3% DSS for three continuous days from PND 0 to PND 3, followed by free access to drinking water until PND 21. The offspring were raised until PND 42. B) PCoA score plots of the gut microbiota in offspring. C, D) Gut microbial compositions at the phylum and genus levels. E) Schematic diagram of APAP‐induced ALF. Offspring from the Ctr and DSS‐treated mother were treated with 300 mg kg^−1^ APAP for 24 h at PND 42 (E‐J). F) Serum ALT and AST levels. G) Serum proinflammatory cytokine levels, including TNF‐α, IL‐6, MCP‐1, and MCP‐3. H) Representative liver images of H&E‐, TUNEL‐, F4/80‐, and Ly6G‐stained livers. I) Liver necrosis area analysis and TUNEL‐positive cell counts. J) F4/80‐ and Ly6G‐positive areas. The data are expressed as the means ± SDs (n = 6). ^*^ *p* < 0.05, ^**^ *p* < 0.01, and ^***^ *p* < 0.001 by one‐way ANOVA followed by Tukey's test (F, G, I, J). Scale bars, 50 µm.

We then investigated whether MGI influences the offspring microbiota in adulthood (**Figure** **2**A). Indeed, offspring from dams with MGI (oDSS) presented different gut microbial alpha diversity compared with offspring from the control dams (oCtr) (Figure S2D, Supporting Information). The oDSS also displayed a different gut microbial structure compared with the oCtr (Figure 2B). At both the phylum and genus levels, the gut microbial compositions of the oDSS and oCtr significantly differed (Figures 2C,D). These results indicate that MGI lastingly impacts the offspring microbiota.

We next investigated the effects of MGI on APAP‐induced acute liver failure (ALF) in offspring (Figure 2E), a serious disease in developed countries that has been reported to be closely associated with the gut microbiota.^[^ ^25^, ^29^ ^]^ Compared with the oCtr group, the oDSS group subjected to APAP had higher serum ALT and AST levels (Figure 2F). Consistently, higher levels of proinflammatory cytokines, including TNF‐α, IL‐6, MCP‐1, and MCP‐3, were detected in the serum of the oDSS than in that of oCtr (Figure 2G). Similarly, oDSS resulted in greater centrilobular necrosis in the livers of APAP‐treated mice than did oCtr, which was confirmed by TUNEL cell death staining (Figures 2H,I). Compared with those in the oCtr group, mice in the oDSS group also presented increased macrophage and neutrophil infiltration in the liver after APAP treatment (Figures 2H,J). Notably, the second offspring from dams with MGI showed similar susceptibility to ALF caused by APAP, as evidenced by their comparable liver damage, immune cell infiltration, and inflammatory responses (Figures S3A–E, Supporting Information). Taken together, these results suggest that MGI lastingly impacts the offspring microbiota and exacerbates ALF in offspring.

### 2.3 MGI‐Mediated Exacerbation of ALF Depends on the Gut Microbiota of Offspring

**Figure 3:**
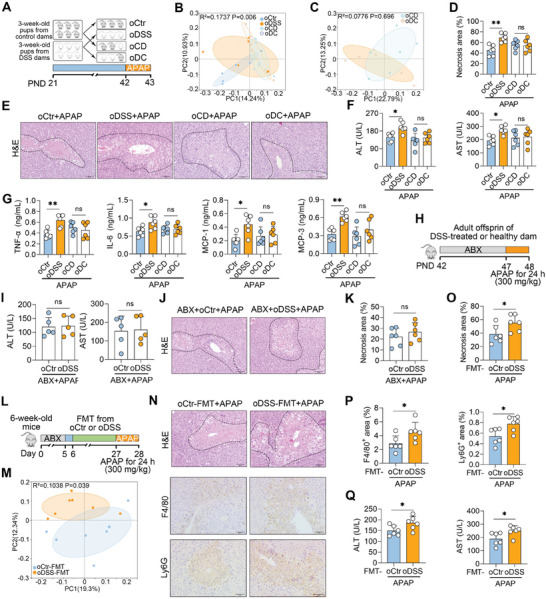
MGI‐mediated exacerbation of ALF depends on the gut microbiota of the offspring. A) Schematic diagram of co‐housing. Three‐week‐old pups from control or DSS dams were placed in a single cage until PND 42, followed by APAP (300 mg kg^−1^) treatment for 24 h. B, C) PCoA score plots of the gut microbiota of cohoused offspring. D, E) Representative H&E‐stained liver images and liver necrosis area analysis. F, G) Serum ALT, AST and proinflammatory indices of cohoused mice. H) Schematic diagram of the depletion of the gut microbiota by antibiotics (ABX). Mice were orally treated with 200 mg kg^−1^ body weight of ampicillin, neomycin, and metronidazole, 100 mg kg^−1^ vancomycin for 5 consecutive days, followed by 300 mg kg^−1^ APAP treatment for 24 h (H–K). I) Serum ALT and AST levels. J, K) Representative H&E‐stained liver images and liver necrosis area analysis. L) Schematic diagram of FMT. Six‐week‐old mice were treated with ABX (200 mg kg^−1^ body weight of ampicillin, neomycin, and metronidazole, 100 mg kg^−1^ vancomycin) for 5 days, followed by FMT for the next 21 days after replacing ABX with water for one day. At day 27, these mice were treated with 300 mg kg^−1^ APAP for 24 h (L–Q). M) PCoA score plots displayed the gut microbial structure from indicated mice. N) Representative images of H&E‐, F4/80‐, and Ly6G‐stained livers. O, P) Liver necrosis and F4/80‐ and Ly6G‐positive area analysis. Q) Serum ALT and AST levels. The data are expressed as the means ± SDs (n = 5–6). ^*^ *p* < 0.05 and ^**^ *p* < 0.01 by one‐way ANOVA followed by Tukey's test (D, F, G) and two‐tailed unpaired Student's t‐test (I, K, O–Q). ns, not significant. Scale bars, 50 µm.

To explore the role of the offspring gut microbiota in MGI‐mediated susceptibility to ALF, co‐housing was performed (**Figure** **3**A). Specifically, offspring with or without MGI were placed in a single cage at weaning to enable a natural exchange of the gut microbiota^[^ ^12^ ^]^ (Figure 3A), and cohoused mice presented similar gut microbial structures and compositions (Figures 3B,C; Figures S3F,G, Supporting Information). Importantly, this normalization of their gut microbiome was associated with similar proneness to APAP‐induced ALF, as evidenced by the equivalent liver injury (Figures 3D–F) and hepatic inflammatory responses (Figure 3G). These results further support the hypothesis that the influence of MGI on the microbiota composition mediates the exacerbation of APAP‐induced ALF in offspring. Notably, the extent of ALF in the cohoused mice was similar to that in the non‐cohoused offspring of the control dams (Figures 3D–G), suggesting a protective role for the commensal microbes that these dams pass along to their offspring.

To confirm the role of the offspring gut microbiota in ALF, offspring aged six weeks were pretreated with antibiotics (ABX) for five days to eliminate their gut microbes, followed by APAP‐induced ALF (Figure 3H). As expected, the serum ALT and AST levels of offspring subjected to MGI and ABX pretreatment (ABX‐oDSS) were comparable to those of ABX‐pretreated control offspring (ABX‐oCtr) (Figure 3I), indicating that the severity of liver damage was similar between the ABX‐oCtr and ABX‐oDSS groups (Figures 3J,K). To explore the extent to which changes in the microbiome resulting from MGI might be sufficient to aggravate ALF, fecal microbiota transplantation (FMT) was performed (Figure 3L). Similar to those from the donor group, FMT from the oDSS group (oDSS‐FMT) resulted in significant differences in the gut microbial structure and composition compared with those from the oCtr group (oCtr‐FMT) (Figure 3M; Figures S3H–J, Supporting Information). As expected, greater liver damage was detected in mice from the oDSS‐FMT group than those in the oCtr‐FMT group (Figures 3N,O). Consistently, the oDSS‐FMT group presented greater hepatic macrophage and neutrophil infiltration than did the oCtr‐FMT group (Figures 3N,P). Similarly, significant increases in the serum ALT, AST, and proinflammatory parameters were observed in the oDSS‐FMT group compared with those in the oCtr‐FMT group (Figure 3Q; Figure S3K, Supporting Information). These results indicate that the MGI‐mediated exacerbation of ALF depends on the gut microbiota of the offspring.

### 2.4 *L. reuteri*‐Derived IAA Ameliorates ALF in Offspring

**Figure 4:**
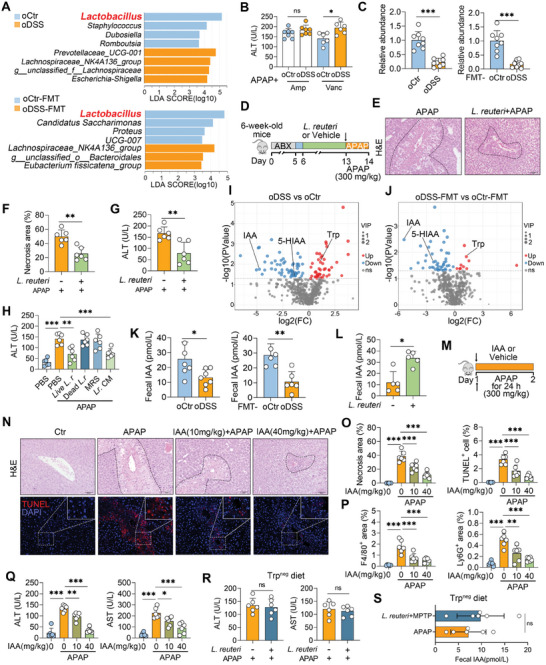
*L. reuteri*‐derived IAA ameliorates ALF in offspring. A) LEfSe (LDA score (log 10) > 3) revealed the differential genus enriched in donor and recipient mice. B) Offspring from different dams were treated with ampicillin (Amp, 100 mg kg^−1^) and vancomycin (Vanc, 50 mg kg^−1^) for five days, followed by APAP treatment, and their serum ALT levels were detected. C) Intestinal *L. reuteri* abundances of different offspring and FMT mice were detected by qPCR. D) Schematic diagram of *L. reuteri* treatment. Mice were treated with ABX for 5 days, after replacing ABX with water for one day, these mice were orally treated with *L. reuteri* (2×10^8^ CFUs/mouse) for 7 days, followed by 300 mg kg^−1^ APAP treatment for 24 h (D–G). E, F) Representative H&E‐stained liver images and liver necrosis area analysis. G) Serum ALT levels from *L. reuteri*‐treated mice. H) Mice were treated with high‐temperature inactivated *L. reuteri* (dead *L. r* group), the control medium (MRS group), and the conditional medium (*L. reuteri*‐cultured supernatant, *Lr*. CM group) in addition to live *L. reuteri*, followed by 300 mg kg^−1^ APAP treatment for 24 h and serum ALT levels were detected. I, J) Volcano map of differentially abundant metabolites from mice in the oCtr and oDSS groups, as well as the corresponding FMT groups. Indole‐3‐acetate (IAA), 5‐hydroxyindoleacetate (5‐HIAA), and tryptophan (Trp) are marked. K, L) Fecal IAA levels in the oCtr and oDSS donor and recipient mice, as well as *L. reuteri*‐treated mice. M) Schematic diagram of IAA treatment. Mice were orally treated with 10 and 40 mg kg^−1^ IAA for 2 h, and then treated with 300 mg kg^−1^ APAP for 24 h (M–Q). N) Representative liver images of H&E‐ and TUNEL‐staining. O) Liver necrosis area analysis and TUNEL‐positive cell counts. P) F4/80‐ and Ly6G‐positive areas. Q) Serum ALT and AST levels of IAA‐treated mice. R, S) Mice treated with *L. reuteri* (2×10^8^ CFUs/mouse) were subjected to a Trp‐deficient diet (Trp^neg^ diet) for 7 days, followed by 300 mg kg^−1^ APAP treatment for 24 h. R) Serum ALT and AST levels. S) Fecal IAA levels. The data are expressed as the means ± SDs (n = 5–8). ^*^ *p* < 0.05, ^**^ *p* < 0.01 and ^***^ *p* < 0.001 by two‐tailed unpaired Student's t‐test (B, C, F, G, K, L, R, S) and one‐way ANOVA followed by Tukey's test (H, O–Q). ns, not significant. Scale bars, 50 µm.

We next sought to examine the underlying mechanism through which susceptibility to ALF is enhanced by microbiota changes in offspring from dams with MGI. LEfSe revealed that the relative abundance of *Lactobacillus* was lower in the oDSS and oDSS‐FMT groups than in the oCtr and oCtr‐FMT groups, respectively (**Figure** **4**A). We first removed *Lactobacilli*, important drivers of indoles with a sensitivity to the antibiotic ampicillin (Amp) and resistance to vancomycin (Vanc),^[^ ^22^, ^31^ ^]^ and found that Amp treatment, but not Vanc treatment, abolished the difference in susceptibility to ALF between mice in the oDSS and oCtr groups (Figure 4B). Moreover, the removal of *Lactobacilli* by Amp in conventional mice aggravated APAP hepatotoxicity (Figure S3L, Supporting Information). At the species level, *Lactobacillus reuteri* (*L. reuteri*) was found to be co‐depleted in mice from the oDSS and oDSS‐FMT groups (Figure S3M, Supporting Information), which was confirmed by qPCR (Figure 4C). We next investigated the effect of *L. reuteri* on APAP‐induced ALF by pretreating mice with *L. reuteri* orally (Figure 4D). Liver damage was alleviated in APAP‐treated mice subjected to *L. reuteri* treatment (Figures 4E,F), which manifested as decreased serum ALT, AST, and inflammatory cytokine levels (Figure 4G; Figures S3N,O, Supporting Information).

To explore whether this function was attributed to *L. reuteri* itself or its metabolic products, we treated mice with *L. reuteri* that had been subjected to high‐temperature inactivation (dead *L. r* group), the control medium (MRS group), and the conditional medium (*L. reuteri*‐cultured supernatant, *Lr*. CM group) in addition to live *L. reuteri*. Interestingly, only the *Lr*. CM group presented a comparable decrease in the serum ALT level relative to that of the live *L. reuteri* group, indicating that the protective effects of *L. reuteri* against ALF were mediated primarily by the molecules secreted by the bacteria (Figure 4H). Next, the fecal metabolic profiles of the mice in the oDSS and oDSS‐FMT groups were significantly altered (Figures S4A–D, Supporting Information). Lower fecal IAA and 5‐HIAA and higher tryptophan (Trp) levels were detected in mice from the oDSS and oDSS‐FMT groups, in which Trp can be metabolized into IAA and 5‐HIAA by *Lactobacillus* ^[^ ^32^ ^]^ (Figures 4I–K; Figure S4E, Supporting Information). In mice treated with *L. reuteri*, increased levels of fecal IAA and 5‐HIAA were also detected (Figure 4L; Figure S4F, Supporting Information). Interestingly, APAP‐treated mice subjected to IAA oral gavage, but not 5‐HIAA, reduced serum ALT levels (Figure S4G, Supporting Information). We further showed that IAA alleviated the liver damage caused by APAP in a dose‐dependent manner (Figures 4M–O). Pretreatment with IAA also reduced APAP‐induced hepatic apoptotic cell, macrophage, and neutrophil infiltration (Figures 4M–P; Figure S4H, Supporting Information). Consistently, IAA treatment reduced APAP‐induced increases in serum ALT, ALT, and proinflammatory cytokine levels in a dose‐dependent manner (Figure 4Q; Figure S4I, Supporting Information). Considering that IAA is derived from Trp, we then confirmed the necessity of Trp in *L. reuteri*‐mediated protection against ALF by feeding mice with a Trp‐deficient diet (Trp^neg^ diet).^[^ ^31^, ^33^ ^]^ Indeed, *L. reuteri* did not reduce the serum ALT and AST levels caused by APAP with a Trp^neg^ diet administration (Figure 4R), which is consistent with the comparable fecal IAA levels between *L. reuteri*‐treated and control mice (Figure 4S). Collectively, these results indicate that *L. reuteri*‐derived IAA ameliorates ALF in offspring.

### 2.5 IAA Alleviates ALF by Promoting IL‐22 Production in the Intestine

**Figure 5:**
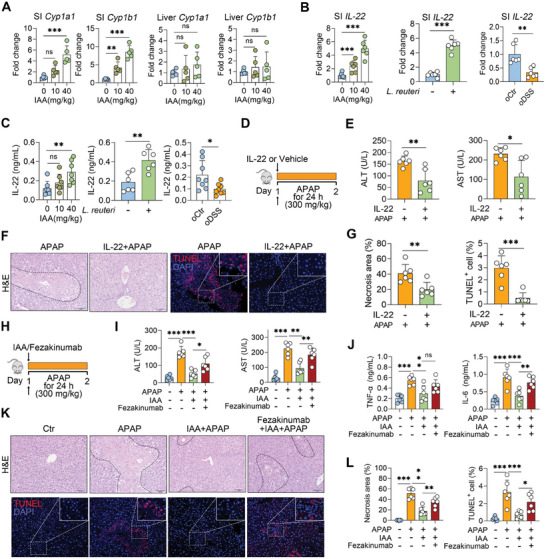
IAA alleviates ALF by promoting IL‐22 production. A) Relative *Cyp1a1* and *Cyp1b1* mRNA expression in the small intestine (SI) and liver of IAA‐treated mice. B) Relative *Il‐22* mRNA expression in the SI of *L. reuteri*‐ and IAA‐treated mice, as well as different offspring. C) Serum IL‐22 levels in *L. reuteri*‐ and IAA‐treated mice and different offspring. D) Schematic diagram of IL‐22 treatment. Mice were intraperitoneally injected with 1 mg kg^−1^ IL‐22 for 2 h, followed by APAP (300 mg kg^−1^) treatment for 24 h. E) Serum ALT and AST levels. F, G) Representative H&E‐ and TUNEL‐stained liver images, liver necrosis area analysis, and TUNEL‐positive cell counts. H) Schematic diagram of IL‐22 signaling blocking by fezakinumab. The mice were intraperitoneally injected with 1 mg kg^−1^ fezakinumab for 1 h before IAA intervention, followed by APAP (300 mg kg^−1^) treatment for 24 h. I, J) Serum ALT, AST, and inflammatory cytokine levels. K) Representative H&E‐ and TUNEL‐stained images. L) Liver necrosis area and TUNEL‐positive cell analysis. The data are expressed as the means ± SDs (n = 5–8). ^*^ *p* < 0.05, ^**^ *p* < 0.01, and ^***^ *p* < 0.001 by one‐way ANOVA followed by Tukey's test (A–C, I, J, L) and two‐tailed unpaired Student's t‐test (C, E, G). ns, not significant. Scale bars, 50 µm.

We next explored the potential mechanism by which IAA alleviated APAP‐induced ALF in mice. First, the mice were administered with IAA by intraperitoneal injection (i.p.) or intragastrically (i.g.), followed by APAP‐induced ALF (Figure S5A, Supporting Information). Interestingly, although both pretreatments with IAA alleviated APAP‐induced ALF, the oral administration of IAA had better protective effects than did the intraperitoneal injection (Figures S5B–D, Supporting Information). Since IAA is known to be an effective agonist of the aryl hydrocarbon receptor (AhR),^[^ ^16^, ^22^, ^34^ ^]^ which regulates host immunity and APAP metabolism,^[^ ^35^ ^]^ we detected the gut and hepatic expression of the *Cyp1a1* and *Cyp1b1* genes, which are regulated by AhR.^[^ ^16^, ^36^ ^]^ Importantly, increased mRNA levels of *Cyp1a1* and *Cyp1b1* were observed in the small intestine (SI), but not in the liver, in mice subjected to IAA (**Figure** **5**A). Moreover, increased expression of interleukin (IL)‐22, an important cytokine involved in tissue repair and regulated by AhR,^[^ ^16^, ^37^ ^]^ in the SI, was observed in IAA‐treated and *L. reuteri*‐treated mice (Figure 5B), whereas lower intestinal IL‐22 expression was detected in offspring subjected to MGI (Figure 5B). These findings were confirmed by the consistent changes in serum IL‐22 levels (Figure 5C). Treating mice with recombinant murine IL‐22 (rMuIL‐22, hereafter referred to as IL‐22) reduces the serum ALT and AST levels (Figures 5D,E), alleviates liver damage (Figures 5F,G), and inhibits hepatic cell apoptosis (Figures 5F,G) and inflammatory responses (Figure S5E, Supporting Information). Blocking IL‐22 with fezakinumab (Figure 5H), a specific antibody against IL‐22,^[^ ^38^ ^]^ weakened the protective role of IAA against APAP‐induced ALF (Figures 5I–L). These results indicate that the IAA alleviates ALF by promoting IL‐22 production in the intestine.

### 2.6 IL‐22 Inhibits Oxidative Stress and Ferroptosis in ALF Mice

**Figure 6:**
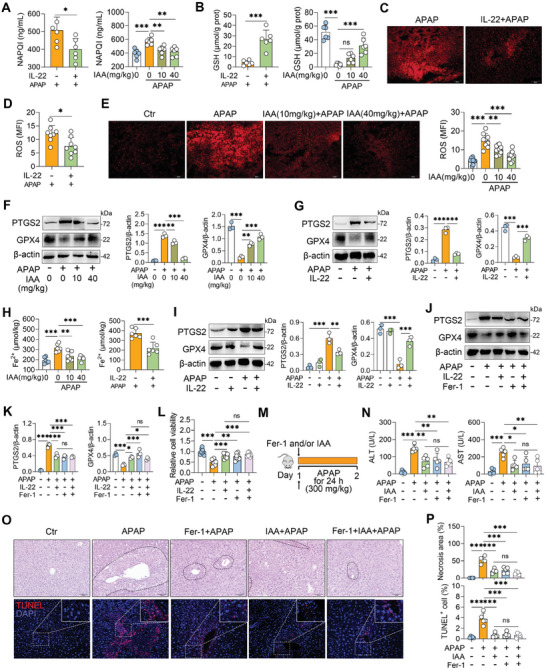
IL‐22 inhibits oxidative stress and ferroptosis in ALF mice. A) The mice were treated with IL‐22 (1 mg kg^−1^) and IAA (40 mg kg^−1^) for 2 h, followed by APAP (300 mg kg^−1^) treatment for 1 h, and hepatic NAPQI levels were detected. B) Hepatic GSH levels from indicated mice. C–E) The mice were treated with IL‐22 (1 mg kg^−1^) and IAA (10 and 40 mg kg^−1^) for 2 h, followed by APAP (300 mg kg^−1^) treatment for 1 h, and liver ROS levels were detected. F, G) Representative hepatic western blot images of IAA‐ and IL‐22‐treated mice. H) Hepatic Fe^2+^ levels of IAA‐ and IL‐22‐treated mice. I) The AML‐12 cells were treated with IL‐22 (0.1 µg mL^−1^) for 2 h, followed by APAP (5mm) treatment for the next 24 h. Representative western blot images of PTGS2 and GPX4 from indicated cells were displayed. J, K) The AML‐12 cells were treated with 1 µm Fer‐1 with or without 0.1 µg mL^−1^ IL‐22 treatment for 2 h, followed by APAP (5mm) treatment for the next 24 h. PTGS2 and GPX4 expressions in indicated cells were detected. L) The AML‐12 cells were treated with 1 µm Fer‐1 with or without 0.1 µg mL^−1^ IL‐22 treatment for 2 h, followed by APAP (5mm) treatment for the next 24 h, and cell viability was detected. M) Schematic diagram of Fer‐1 and IAA treatment. The mice were treated with Fer‐1 (10 mg kg^−1^) with or without IAA (40 mg kg^−1^) for 2 h, followed by APAP (300 mg kg^−1^) treatment for 24 h. N) Serum ALT and AST levels. O) Representative H&E‐ and TUNEL‐stained images. P) Liver necrosis area and TUNEL‐positive cell analysis. The data are expressed as the means ± SDs. ^*^ *p* < 0.05, ^**^ *p* < 0.01, and ^***^ *p* < 0.001 by one‐way ANOVA followed by Tukey's test (A, B, E–L, N, P) and two‐tailed unpaired Student's t‐test (A, B, D). ns, not significant. Scale bars, 50 µm.

Three key stages are involved in the pathogenesis of APAP‐induced ALF: APAP absorption, NAPQI formation, and hepatic regeneration.^[^ ^25^, ^29^ ^]^ We detected no significant difference in the area under the curve (AUC) in mice pretreated with IAA or IL‐22 (Figures S6A,B, Supporting Information). Moreover, the protective effect of IAA pretreatment was unlikely to modulate hepatic regeneration, which manifested as a stable expression of proliferating cell nuclear antigen (PCNA) in mice pretreated with IAA and IL‐22 compared with APAP treatment (Figures S6C,D, Supporting Information). Consistent with the stable hepatic expression of *Cyp1a1* and *Cyp1b1*, IAA and IL‐22 treatments did not alter the hepatic *Cyp2e1* levels (Figures S6E,F, Supporting Information), suggesting that the IAA‐induced alleviation of ALF was not the result of suppressed APAP bioactivation. However, the NAPQI levels were reduced in APAP‐treated mice subjected to IAA and IL‐22 treatments (**Figure** **6**A). Similarly, APAP‐treated mice in the oDSS group presented increased NAPQI levels (Figure S6G, Supporting Information). GSH can neutralize NAPQI to prevent host oxidative damage;^[^ ^25^, ^29^ ^]^ thus, we hypothesized that the reduction in NAPQI by IAA and IL‐22 administration was regulated via GSH production. As expected, increased hepatic GSH levels, the GSH/GSSG ratios, and other hepatic antioxidants, including SOD and CAT, were observed in the mice pretreated with IL‐22 and IAA (Figure 6B; Figures S6H,I, Supporting Information). Moreover, we found that IAA and IL‐22 inhibited APAP‐induced hepatic ROS accumulation (Figures 6C–E). Furthermore, the administration of IL‐22 reversed the decreases in T‐GSH, GSH, GSH/GSSH, SOD, and CAT caused by APAP in vitro (Figure S6J, Supporting Information). These antioxidant enzymes were also reduced in APAP‐treated offspring subjected to MGI (Figure S6K, Supporting Information). In addition, the administration of IAA and IL‐22 also protected mice against ethanol‐induced ALF (Figures S6L,M, Supporting Information), which is another important cause of ALF caused by excessive oxidative stress.^[^ ^39^ ^]^

We next found that APAP‐treated mice presented reduced GPX4 and increased PTGS2 levels in the liver, while IAA and IL‐22 pretreatment reversed these alterations (Figures 6F,G). Increased hepatic Fe^2+^ accumulation was detected in mice after APAP treatment, and this increase was reversed by IAA or IL‐22 administration (Figure 6H). We also confirmed that APAP‐treated offspring subjected to MGI presented greater Fe^2+^ accumulation and PTGS2 expression than did the oCtr group (Figures S7A,B, Supporting Information)*. In vitro*, pretreatment with IL‐22 alleviated the APAP‐induced increase in PTGS2 and restored GPX4 expression in hepatocytes (Figure 6I).

We next showed that both IL‐22 and ferrostatin‐1 (Fer‐1) inhibited ferroptosis and restored the decrease in cell viability caused by APAP, but no further influence was detected in Fer‐1 and IL‐22 co‐treated cells (Figures 6J–L). We then administered Fer‐1 to IAA‐treated mice. Interestingly, although both IAA and Fer‐1 reduced APAP‐induced serum ALT and AST levels and liver damage, no additional effect was detected in Fer‐1‐treated mice subjected to IAA administration (Figures 6M–P; Figure S7C, Supporting Information). Both IAA and Fer‐1 reduced hepatic PTGS2 and increased the GPX4 levels, but these alternations were stable upon IAA and Fer‐1 co‐stimulations (Figure S7D, Supporting Information). These findings were also confirmed by the Fe^2+^ levels (Figure S7E, Supporting Information). Collectively, these results indicate that IAA‐mediated IL‐22 production inhibits oxidative stress and ferroptosis in ALF mice.

### 2.7 IL‐22 Alleviates Ferroptosis and ALF by Activating STAT3

**Figure 7:**
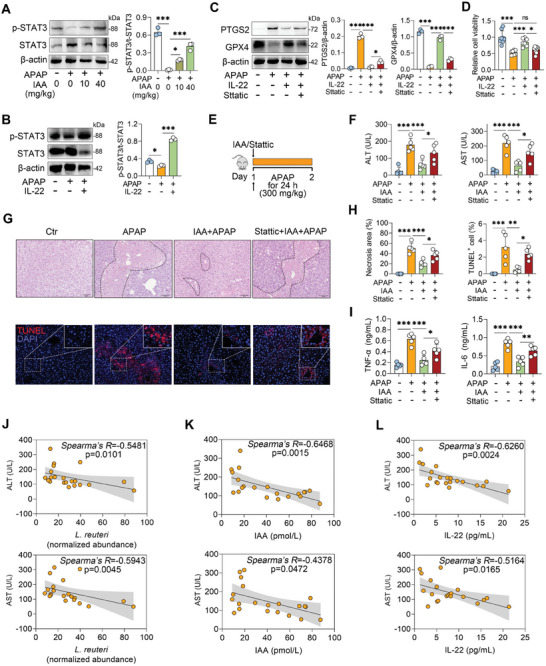
IL‐22 alleviates ferroptosis and ALF by activating STAT3. A, B) Representative western blot images of p‐STAT3 and STAT3 from IAA‐ and IL‐22 treated mice. C, D) The cells were treated with 20 µm Stattic for 1 h, and then treated with IL‐22 (0.1 µg mL^−1^) for 2 h, followed by APAP (5mm) treatment for the next 24 h. PTGS2 and GPX4 expressions (C) and cell viability (D) were detected. E) Schematic diagram of STAT3 inhibition by Stattic. The mice were intraperitoneally injected with 10 mg kg^−1^ Stattic for 1 h before IAA (40 mg kg^−1^) intervention, followed by APAP (300 mg kg^−1^) treatment for 24 h. F) Serum ALT and AST levels. G) Representative H&E‐ and TUNEL‐stained images. H) Liver necrosis area and TUNEL‐positive cell analysis. I) Serum proinflammatory cytokine levels. J) Correlations between the normalized abundance of *L. reuteri* in feces and the serum ALT or AST levels of ALF patients. The relative abundance of *L. reuteri* was quantified via normalization of its absolute quantity relative to that of the total 16S rRNA gene in the fecal samples using the comparative CT method. n = 20. K) Correlations between the intestinal IAA concentration and the serum ALT or AST level in ALF patients. n = 20. L) Correlations between the serum IL‐22 concentration and the serum ALT or AST level in ALF patients. n = 20. The data are expressed as the means ± SDs. ^*^ *p* < 0.05, ^**^ *p* < 0.01, and ^***^ *p* < 0.001 by one‐way ANOVA followed by Tukey's test (A‐D, F, H, I). ns, not significant. Scale bars, 50 µm.

The signal transducer and activator of transcription 3 (STAT3) is the downstream target of IL‐22 and has been linked to the inhibition of ferroptosis via the upregulation of GPX4.^[^ ^37^, ^40^ ^]^ As expected, mice treated with APAP presented reduced phosphorylation (p)‐STAT3 levels in the liver, whereas IAA and IL‐22 treatments restored hepatic p‐STAT3 expression (**Figures** **7**A,B). In vitro, the inhibition of STAT3 by Stattic weakened the inhibitory effect of IL‐22‐mediated protection against APAP‐induced ferroptosis (Figures 7C,D). We next administered IAA‐treated mice with Stattic and reported that the protective effects of IAA against APAP hepatotoxicity were also weakened by Stattic, as evidenced by increased serum ALT and AST levels (Figures 7E,F), liver damage (Figures 7G,H), and inflammatory responses (Figure 7I) upon STAT3 inhibition compared with those in mice treated with IAA alone. Similarly, STAT3 inhibition abolished the inhibitory effect of IAA treatment on APAP‐induced Fe^2+^ accumulation (Figure S7F, Supporting Information). In addition, the abundance of *L. reuteri* was negatively correlated with the serum ALT and AST levels in patients with ALF (Figure 7J). Moreover, the fecal IAA and serum IL‐22 levels were inversely associated with the serum concentrations of ALT and AST in ALF patients (Figures 7K,L). Taken together, these results demonstrate that MGI reduces *L. reuteri*‐derived IAA in offspring, which alleviates ALF by inhibiting STAT3‐mediated ferroptosis through the promotion of IL‐22 production.

## 3 Discussion

Microbial transmission from mother to offspring plays an important role in the development of the gut microbiota and immunity of offspring. Disruption of these processes may thus affect immune status and disease outcomes. During critical prenatal periods, maternal microbial metabolites, such as trimethylamine‐N‐oxide and 3‐indoxyl sulfate, promote fetal neurodevelopment in healthy mice.^[^ ^41^ ^]^ In contrast, maternal immune activation‐derived gut dysbiosis facilitates neurodevelopmental abnormalities in offspring through the production of IL‐17a.^[^ ^42^ ^]^ In addition, changing the diet pattern by enhancing the fat content and depriving the offspring of fiber during pregnancy promoted the development of cognitive impairment and respiratory infection in the offspring.^[^ ^11^, ^43^ ^]^ Interestingly, unlike neurodevelopmental disorders, which are often determined prenatally, studies have shown that increased susceptibility to diseases in offspring caused by maternal immune activation or maternal diet alteration might be postnatally determined.^[^ ^13^, ^43^ ^]^ However, it is still unclear whether and how MGI during lactation influences gut microbiota and disease susceptibility in offspring.

Breastfeeding is one of the predominant factors for shaping the gut microbiota of offspring. Compared with nonexclusively breastfed infants, exclusively breastfed infants presented lower relative abundances of *Bacteroidetes* and *Firmicutes* and higher relative abundances of pathways related to lipid metabolism, vitamin metabolism, and detoxification.^[^ ^44^ ^]^ Breastfeeding duration impacts the retention and functional diversity of the *Bifidobacterium longum* strain.^[^ ^45^ ^]^ However, maternal immune status directly affects the composition of progeny microflora through breast milk. For example, maternal fiber deprivation alters the microbiota in offspring, resulting in low‐grade inflammation and a predisposition to obesity.^[^ ^12^ ^]^ In the present study, we demonstrated that gut inflammation during lactation significantly changed the gut and mammary microbial structures and compositions. A previous study revealed that infants born to mothers with inflammatory bowel disease (IBD) presented an altered gut microbiota, especially a reduced abundance of *Bifidobacterium*.^[^ ^46^ ^]^ Interestingly, gut inflammation progressively increased the abundance of *Firmicutes* in the gut and mammary glands, whereas *Bacteroidota* and *Proteobacteria* showed different trends in the gut and mammary glands. In non‐lactating mice, increased *Firmicutes* and *Proteobacteria* and reduced *Bacteroidota* abundances were also observed.^[^ ^47^ ^]^ These changes are associated with increased mammary inflammatory responses, which is consistent with previous studies showing that gut dysbiosis contributes to the development of mastitis and mammary dysbiosis.^[^ ^48^, ^49^, ^50^ ^]^ Interestingly, the kinetics of DSS‐induced mastitis are the same as those in the colon, which implies that gut inflammation is an effective trigger for mastitis and indicates potential microbial translocation. In addition to direct microbial translocation, gut inflammation‐induced barrier damage may result in the translocation of harmful factors, including LPS, into the mammary gland, leading to altered mammary microbiota.^[^ ^51^ ^]^ Gut dysbiosis‐derived LPS can also damage the blood‐milk and vascular barriers and thus facilitate mammary dysbiosis and inflammation.^[^ ^30^, ^50^, ^51^ ^]^ Consistently, a previous study also showed that gut inflammation induced kinetics similar to those associated with damage to the blood‐milk barrier.^[^ ^30^ ^]^

Similar to maternal diet‐induced alterations in the gut microbiota of offspring,^[^ ^12^ ^]^ we found that MGI during early lactation influenced the gut microbiota of offspring until adulthood, resulting in aggravated ALF caused by APAP. This observation highlights that early life is a window for shaping the adult gut microbiota and regulating disease tolerance. Indeed, dysbiosis of early‐life gut microbiota, such as that caused by antibiotic exposure, induces continuous microbial dysbiosis and impairs natural killer cell maturation, intestinal stem cell differentiation, and macrophage polarization.^[^ ^52^, ^53^ ^]^ Notably, we found a reduced relative abundance of *Lactobacillus* in offspring with MGI. Consistently, it has been reported that early‐life gut dysbiosis reduces *Lactobacillus* abundance, resulting in aggravated necrotizing enterocolitis by impairing gut stem cell niches.^[^ ^53^ ^]^ Another study showed that antibiotic‐induced deprivation of *Lactobacillus* in early life exacerbated obesity by reducing the activation of gut PPAR‐γ.^[^ ^54^ ^]^ As a potential probiotic, a decrease in the abundance of *Lactobacillus* in the gut has also been associated with exacerbated ALF by reducing the production of beneficial metabolites, including daidzein and 5‐methoxyindoleacetic acid, in a species‐specific manner.^[^ ^25^, ^39^ ^]^ Importantly, numerous studies have shown that *Lactobacillus* species are well‐known producers of AhR ligands derived from dietary Trp.^[^ ^15^, ^17^, ^22^ ^]^ We found that offspring with MGI had disrupted microbial Trp metabolism via a decrease in fecal IAA and 5‐HIAA levels but an increase in Trp levels, which was associated with aggravated ALF. We next confirmed that treatment with IAA alleviated APAP‐induced ALF, which is consistent with previous studies showing that IAA plays a protective role in liver injury caused by monocrotaline or ethanol,^[^ ^16^, ^55^ ^]^ as well as in LPS‐ or cytokine‐induced liver inflammation.^[^ ^56^ ^]^

Endogenous ligands, including IAA‐mediated AhR activation, have been reported to protect mice against liver injury caused by ethanol.^[^ ^16^, ^55^, ^57^ ^]^ In contrast, AhR activation in the liver promoted CYP2E1 expression and subsequent NAPQI production, leading to exacerbated APAP hepatotoxicity.^[^ ^35^ ^]^ However, we found that treating with IAA induced AhR activation in the gut but not in the liver, which is consistent with the findings of previous studies showing that oral gavage with IAA did not cause systemic AhR activation.^[^ ^16^ ^]^ Moreover, treating with IAA did not influence liver CYP2E1 expression. These findings indicate that the protective effects of IAA against APAP‐induced ALF are unlikely to activate hepatic AhR. We next showed that treating with IAA facilitated IL‐22 production and that the administration of IL‐22 alleviated ALF, whereas blocking IL‐22 weakened the protective effects of IAA against APAP hepatotoxicity. As a main cytokine for tissue repair, IL‐22 has been widely reported to alleviate multiple types of tissue injury and restore barrier function.^[^ ^36^, ^37^ ^]^ In addition, increased IL‐22 was also associated with improved liver injury.^[^ ^16^ ^]^ Interestingly, IAA‐induced IL‐22 did not affect APAP absorption or hepatic regeneration but limited excessive oxidative stress caused by APAP.

Excessive oxidative stress is increasingly associated with ferroptosis during APAP‐induced ALF, in which GPX4 is commonly reduced and subsequently promotes lipid peroxidation.^[^ ^25^, ^58^ ^]^ We further found that *L. reuteri*‐ and IAA‐induced IL‐22 release reversed the decrease in GPX4 and ameliorated ferroptosis caused by APAP. Previous studies have also found that *L. reuteri* and IAA have effective antioxidant capacity.^[^ ^39^, ^59^ ^]^ After binding with its receptor, IL‐22 primarily induces STAT3 activation, promoting cell proliferation and local tissue regeneration.^[^ ^37^, ^60^, ^61^ ^]^ Recently, STAT3 has been reported to inhibit ferroptosis by binding to the promoters of genes associated with negative ferroptosis regulation, including GPX4, SLC7A11, and FTH1.^[^ ^40^, ^62^ ^]^ Indeed, the inhibition of STAT3 weakened the effects of IAA and IL‐22 on APAP‐induced ferroptosis and hepatotoxicity. Similarly, another study confirmed that the protective effects of IL‐22 in APAP‐induced ALF depend on the activation of STAT3.^[^ ^63^ ^]^

Collectively, our results revealed that MGI during early lactation significantly altered the gut and mammary microbiomes of lactating mice. MGI during early lactation strongly influences the gut microbiota of dams and pups at weaning, which lastingly affects the offspring's gut microbiota and metabolism in adulthood, resulting in exacerbated APAP hepatotoxicity. Moreover, supplementing with the microbial metabolite IAA alleviated APAP‐induced ALF in mice through the inhibition of ferroptosis via the IL‐22‐STAT3 axis. Our findings indicate that MGI during lactation shapes gut microbial metabolism and alters tolerance to ALF in offspring, highlighting the important role of maintaining normal microbial transmission in shaping the offspring microbiota and providing a potential strategy for intervention in ALF through the regulation of microbial metabolism.

## 4 Experimental Section

### Animal

The animal experiments were approved by the Institutional Animal Care and Use Committee (IACUC) of Jilin University (SY202412015). Specific pathogen‐free (SPF)‐grade C57BL/6J mice (6–8 weeks old) were purchased from Liaoning Changsheng Biotechnology Co. Ltd. (Benxi, China). All the mice were housed on 12 h light/dark cycles with free access to food and water.

### Human Samples

The fecal and serum samples from patients with ALF were obtained from China‐Japan Union Hospital of Jilin University. Informed consent was obtained from all participants, and the experimental protocol was approved by the Institutional Ethical Committee.

### Maternal Gut Inflammation Model

A total of 240 female and male mice (C57BL/6, 6–8 weeks old) were caged at a ratio of 3:1 for 24 h, and pregnancy was confirmed through vaginal plugs. Male mice were then removed, and female mice were housed until parturition. At postnatal day (PND)0, these mice (n = 5–8) were treated with 3% DSS in drinking water for three consecutive days, followed by drinking water only for the next 2 days (PND3 to PND5).^[^ ^30^ ^]^ The mice were sacrificed at the indicated time points (PND0, PND1, PND3, and PND5), and fecal, serum, mammary gland, colon, and liver samples were collected aseptically and stored at −80 °C for detection as previously described.^[^ ^48^ ^]^ Fecal samples from dams and pups at PND21 (weaning) in another parallel experiment were also collected, and these pups were housed until PND42 (6 weeks old) for fecal microbiota analysis and detection of susceptibility to liver damage. Moreover, the dams were then mated with male mice at PND21 to generate second offspring, which were housed until they were 6 weeks old for the animal experiments.

### APAP‐Induced Acute Liver Failure Model

Male mice (n = 6, 6–8 weeks old) were fasted for 12 h with free access to water, and the mice were then orally treated with 300 mg kg^−1^ APAP at 8:00 PM for 24 h. For *L. reuteri* treatment, mice were treated with an antibiotics cocktail (ABX, 200 mg kg^−1^ body weight of ampicillin, neomycin, and metronidazole, 100 mg kg^−1^ vancomycin) for five consecutive days to remove commensal microbes, after replacing ABX for one day, these mice were orally treated with *L. reuteri* (2×10^8^ CFUs/mouse) for 7 days, followed by 300 mg kg^−1^ APAP treatment for 24 h. To explore the role of tryptophan metabolism in the protective effects, the *L. reuteri*‐mice was also treated with a Trp‐deficient diet (Trpneg diet) as previously described.^[^ ^64^ ^]^ For IAA treatment, the mice (n = 6) were pretreated with 10 or 40 mg kg^−1^ IAA via oral administration for 2 h. For IL‐22 signaling and STAT3 inhibition, the mice (n = 6) were intraperitoneally injected with 1 mg kg^−1^ fezakinumab (anti‐IL‐22) and 10 mg kg^−1^ Stattic for 1 h before IAA intervention. For ferroptosis inhibition, the mice were treated with ferrostatin‐1 (Fer‐1, 10 mg kg^−1^) with or without IAA. For IL‐22 intervention, the mice were intraperitoneally injected with 1 mg kg^−1^ IL‐22 for 2 h, followed by APAP treatment. For the detection of hepatic reactive oxygen species (ROS) and NAPQI, hepatic tissues were collected 1 h after APAP (300 mg kg^−1^) treatment. For the assay of APAP absorption, plasma samples were collected at 0, 15, 45, 90, and 120 min after APAP (300 mg kg^−1^) treatment. Other samples were collected at 24 h after APAP (300 mg kg^−1^) treatment.

### Ethanol‐Induced ALF Model

Mice were fasted for 12 h with free access to water, and then treated with 6 mg kg^−1^ ethanol by oral administration for 6 h.^[^ ^39^ ^]^

### Antibiotic Treatment and Fecal Microbiota Transplantation (FMT) Model

For the elimination of the gut microbiota, mice were orally treated with an antibiotics cocktail (200 mg kg^−1^ body weight of ampicillin, neomycin, and metronidazole, 100 mg kg^−1^ vancomycin) for five consecutive days. FMT experiment was performed as previously,^[^ ^48^, ^50^ ^]^ fresh fecal samples from offspring (6 weeks old) with or without maternal gut inflammation were collected aseptically, and samples from the same group were mixed and dissolved in sterile PBS with a final concentration of 100 mg mL^−1^. Recipient male mice (6 weeks old) were depleted of the commensal microbes as mentioned above, after replacing antibiotics with water for one day, these mice were orally administrated with fecal supernatant from offspring fed by dams with (oDSS) or without (oCtr) gut inflammation. FMT was first performed for three consecutive days, and then once every two days for a total of 3 weeks.

### Co‐Housing Experiment

Co‐housing experiment was performed as previously.^[^ ^12^ ^]^ In detail, male pups from dams with (oDSS) or without (oCtr) gut inflammation during lactation were weaned at 3 weeks old, oDSS and oCtr were co‐housed in single cages with non‐co‐housed siblings serving as controls. These cohoused and non‐co‐housed siblings were housed until 6 weeks of age and then performed for APAP‐induced ALF.

### Cell Culture and Treatment

Alpha mouse liver 12 (AML‐12) cells were purchased from American Type Culture Collection (ATCC, VA, USA) and cultured in DMEM/F‐12 (1:1) medium containing 1% insulin‐transferrin‐selenium solution (Sigma–Aldrich, MO, USA), 10% fetal bovine serum (Gibco, CA, USA), and 40 ng mL^−1^ dexamethasone (Sigma–Aldrich, MO, USA) in a suitable incubator at 37 °C with 5% CO_2_. For IL‐22 treatment, the cells were treated with IL‐22 (0.1 µg mL^−1^) for 2 h, followed by APAP (5 mm) treatment for the next 24 h. For Fer‐1 treatment, the AML‐12 cells were treated with 1 µm Fer‐1 with or without 0.1 µg mL^−1^ IL‐22 treatment for 2 h, followed by APAP (5 mm) treatment for the next 24 h. For the inhibition of STAT3, the cells were treated with 20 µm Stattic for 1 h, and then treated with IL‐22 (0.1 µg mL^−1^) for 2 h, followed by APAP (5 mm) treatment for the next 24 h.

### Bacterial Culture

*L. reuteri* was isolated and verified using 16S rRNA sequencing as previously described.^[^ ^33^ ^]^ *L. reuteri* was cultured in MRS (Hopebio, Qingdao, China) broth with 0.05% L‐cysteine at 37 °C anaerobic conditions for 48 h as previously described.^[^ ^65^ ^]^ Bacteria were heated in the 100 °C metal bath for 2 h to acquire dead *L. reuteri*.

### Histological Analysis

Tissues used for histological analysis including liver, mammary glands, and colon were fixed with 4% paraformaldehyde for more than 48 h, followed by paraffin embedding and preparing for 5 µm slices. The prepared slices were then stained with hematoxylin & eosin (H&E) and analyzed using an optical microscope (Olympus, Tokyo, Japan). Histological scores or hepatic necrosis area were determined as previously described.^[^ ^25^, ^48^ ^]^

### Immunohistochemistry

Tissues were collected and prepared for 5 µm paraffin sections. Sections were dewaxed and hydrated with xylene and gradient alcohol. For hepatic macrophage and neutrophils detection, immunohistochemistry was performed using SAP (Mouse/Rabbit) IHC Kit (MXB, China) as mentioned previously.^[^ ^48^ ^]^ In brief, the prepared sections were treated with endogenous peroxidase blockers for 40 min at room temperature (RT). After washing with PBS three times per 5 min nonimmune goat serum was administrated for 40 min at RT. Furthermore, these sections were stained with F4/80 (Cell Signaling Technology, MA, USA) and Ly6G (Cell Signaling Technology, MA, USA) overnight at 4 °C. After washing with PBS, goat‐anti‐rabbit IgG was administrated for 30 min at RT, followed by horseradish peroxidase incubation for 20 min. Moreover, sections were treated with color developing agent for 5 min and nuclei were stained with hematoxylin for 2 min. After differentiation by 1% muriatic acid alcohol, ammonium hydroxide treatment, and dehydration, these sections were mounted utilizing neutral resins and detected using an optical microscope (Olympus, Tokyo, Japan).

### Immunofluorescence

For hepatic apoptotic cell assay, dewaxed and hydrated sections were stained with a commercial terminal deoxynucleotidyl transferase dUTP nick end labeling (TUNEL) assay kit and detected in confocal fluorescence microscopy (Olympus, Tokyo, Japan). Apoptotic cells were labeled in red and analyzed by ImageJ software.^[^ ^25^ ^]^

### Determination of ROS

For hepatic ROS detection, hepatic tissues were collected and prepared for frozen sections. Frozen liver sections were treated with dihydroethidium (DHE, Thermo Scientific, MA, USA) for 20 min and ROS level was immediately detected by Fluorescence microscope (Olympus, Tokyo, Japan) and analyzed using ImageJ software as previously mentioned.^[^ ^25^ ^]^

### Biochemical and ELISA Assays

For the detection of serum alanine aminotransferase (ALT) and aspartate aminotransferase (AST) levels detections, commercial ALT (Nanjing Jiancheng Bioengineering Institute, Nanjing, China) and AST (Nanjing Jiancheng Bioengineering Institute, Nanjing, China) assay kits were used. Commercial ELISA kits including TNF‐α, IL‐1β, MCP‐1, and MCP‐3 were used to analyze serum proinflammatory cytokines levels. For proinflammatory cytokines assay of tissues, TNF‐α (Biolegend, CA, USA), IL‐1β (Biolegend, CA, USA), and IL‐6 (Biolegend, CA, USA) ELISA kits were also used. For antioxidant analysis, commercial GSH (Nanjing Jiancheng Bioengineering Institute, Nanjing, China), GSSG (Nanjing Jiancheng Bioengineering Institute, Nanjing, China), CAT (Nanjing Jiancheng Bioengineering Institute, Nanjing, China), and SOD (Nanjing Jiancheng Bioengineering Institute, Nanjing, China) assay kits were performed. NAPQI (Boshen, Nanjing, China) kit was performed for hepatic NAPQI detection according to the manufacturer's instructions.

### Cell Viability Assay

Cell viability was analyzed by a commercial CCK8 assay kit (Solarbio, Bejing, China) according to the manufacturer's instructions.

### Assay of Cellular Iron

Cellular Fe^2+^ level was determined by commercial kit according to the manufacturer's instructions (E‐BC‐F101, Elabscience, China).

### Assay of APAP

APAP was detected by high‐performance liquid chromatography as previously.^[^ ^25^ ^]^ Briefly, plasma samples were collected and extracted with methanol and centrifuged at 16,000 g for 15 min. The supernatant was processed by vacuum drying and reconstituted in methanol. A total volume of 10 mL sample was injected into a Hypersil ODS (C18) column (Thermo Scientific, MA, USA). The mobile phase was methanol and water at a volume of 50:50. Data were acquired and analyzed using Agilent LC1260 software.

### RNA Extraction and Quantitative Reverse Transcription PCR (qRT‐PCR)

Total RNA was extracted using a TRIzol reagent (Invitrogen, CA, USA) and the cDNA was synthesized using a reverse transcription reagent kit (TransGen Biotech, Beijing, China). A FastStart Universal SYBR Green Master Mix (ROX) (Roche, Switzerland, Basel) was used for qRT‐PCR on a 7500 Real‐Time PCR System (Applied Biosystems, CA, USA). Specific primer sequences were as follow: *Ptgs2* (sense 5′‐TTCCAATCCATGTCAAAACCGT‐3′, antisense 5′‐AGTCCGGGTACAGTCACACTT‐3′), *Cyp1a1* (sense 5′‐CTCTTCCCTGGATGCCTTCAA‐3′, antisense 5′‐GGATGTGGCCCTTCTCAAATG‐3′), *Cyp1b1* (sense 5′‐AATGAGGAGTTCGGGCGCACA‐3′, antisense 5′‐GGCGTGTGGAATGGTGACAGG‐3′), *Il22* (sense 5′‐CATGCAGGAGGTGGTACCTT‐3′, antisense 5′‐CAGACGCAAGCA TTTCTCAG‐3′), *Cyp2e1* (sense 5′‐CGTTGCCTTGCTTGTCTGGA‐3′, antisense 5′‐AAGAAAGGAATTGGGAAAGGTCC‐3′), *L. reuteri* (sense 5′‐GCCGCCTAAGGTGGGACAGAT‐3′, antisense 5′‐AACACTCAAGGATTGTCTGA‐3′), *16S* (sense 5′‐TGATGCACTTGCAGAAAACA‐3′, antisense 5′‐ACCAGAGGAAATTTTCAATAGGC‐3′), *GAPDH* (sense 5′‐AACTTTGGCATTGTGGAAGG‐3′, antisense 5′‐ACACATTGGGGGTAGGAACA‐3′). GAPDH and 16S served as endogenous controls for host or bacteria, respectively.

### Protein Extraction and Western Blotting

Tissue and cell samples were lysed by a tissue protein extract (Thermo Fisher Scientific, MA, USA) and the protein concentration was detected using a BCA kit (Thermo Scientific, MA, USA). Targeted proteins were separated by SDS‐PAGE and transferred into PVDF membranes, followed by blocking with 5% skim milk for 3 h at RT. The specific primary antibodies were as follows: PTGS2 (Affinity Biosciences, NJ, USA), GPX4 (Affinity Biosciences, NJ, USA), STAT3 antibody (Affinity Biosciences, NJ, USA), phospho‐STAT3 (Thr705, Affinity Biosciences, NJ, USA), PCNA antibody (Cell Signaling Technology, MA, USA), β‐actin antibody (Affinity Biosciences, NJ, USA). All the primary antibodies were incubated at 4 °C overnight followed by Goat anti‐rabbit or Rabbit anti‐mouse IgG (1:20,000) and determined using the ECL plus western blotting Detection System (Tanon, China). The expression level of the target protein was quantitatively analyzed by ImageJ.

### Total Bacterial DNA Extraction and 16S rRNA Gene Sequencing Analysis

Mammary tissues used for 16S rRNA sequencing were collected aseptically. In brief, mice were anesthetized and euthanized, and the skin on the nipple surface was disinfected with alcohol. Under sterile conditions, the skin of the abdomen of the mice was cut and the mammary tissue was collected as previously.^[^ ^48^, ^49^, ^66^ ^]^ Microbial community genomic DNA was extracted from mouse fecal samples and mammary glands using the FastDNA Spin Kit for Soil (MP Biomedicals, USA) according to the manufacturer's instructions. The DNA extract was checked on 1% agarose gel, and DNA concentration and purity were determined with a NanoDrop 2000 UV–vis spectrophotometer (Thermo Fisher Scientific, MA, USA). The hypervariable region V3‐V4 of the bacterial 16S rRNA gene was amplified with primer pairs 338F (5′‐ACTCCTACGGGAGGCAGCAG‐3′) and 806R (5′‐GGACTACHVGGGTWTCTAAT‐3′) by an ABI GeneAmp 9700 PCR thermocycler (ABI, CA, USA). The PCR amplification of the 16S rRNA gene was performed as mentioned previously.^[^ ^49^ ^]^ PCR reactions were performed in triplicate and the PCR product was extracted from 2% agarose gel and purified using the AxyPrep DNA Gel Extraction Kit (Axygen Biosciences, CA, USA) according to manufacturer's instructions and quantified using Quantus Fluorometer (Promega, USA). Purified amplicons were pooled in equimolar and paired‐end sequenced on an Illumina MiSeq PE300 platform/NovaSeq PE250 platform (Illumina, San Diego, USA) by Majorbio Bio‐Pharm Technology Co. Ltd. (Shanghai, China). OTUs with 97% similarity cutoff were clustered using UPARSE version 7.1, and chimeric sequences were identified and removed. The taxonomy of each OTU representative sequence was analyzed by RDP Classifier version 2.2 against the 16S rRNA database using a confidence threshold of 0.7. Principal coordinate analysis (PCoA) was used to identify microbial structure and linear discriminant analysis effect size (LEfSe) was performed to identify bacterial taxa that were differentially enriched in different treatment groups.

### Untargeted Metabolomics

Fecal samples (0.15 mg) were weighted and added 600 µL MeOH (Containing 2‐Amino‐3‐(2‐chloro‐phenyl)‐propionic acid (4 ppm) in a 2 mL centrifuge tube, vortex for 30 s and placed in a tissue grinder with steel balls for 120 s at 50 Hz, followed by room temperature ultrasound for 10 min and centrifuged at 15,000 rpm, 4 °C for 10 min. The supernatant was filtered by 0.22 µm membrane and transferred into the detection bottle for LC‐MS detection. The LC analysis was performed on a Vanquish UHPLC System (Thermo Fisher Scientific, MA, USA). Chromatography was carried out with an ACQUITY UPLC ® HSS T3 (2.1× 100 mm, 1.8 µm) (Waters, Milford, MA, USA). The column maintained at 40 °C. The flow rate and injection volume were set at 0.3 mL min^−1^ and 2 µL, respectively. For LC‐ESI (+)‐MS analysis, the mobile phases consisted of (B2) 0.1% formic acid in acetonitrile (v/v) and (A2) 0.1% formic acid in water (v/v). Separation was conducted under the following gradient: 0–1 min, 8% B2; 1–8 min, 8%–98% B2; 8–10 min, 98% B2; 10–10.1 min, 98%–8% B2; 10.1–12 min, 8% B2. For LC‐ESI (‐)‐MS analysis, the analytes were carried out with (B3) acetonitrile and (A3) ammonium formate (5 mm). Separation was conducted under the following gradient: 0–1 min, 8% B3; 1–8 min, 8%–98% B3; 8–10 min, 98% B3; 10–10.1 min, 98%–8% B3; 10.1–12 min, 8% B3. Mass spectrometric detection of metabolites was performed on Q Exactive Focus (Thermo Fisher Scientific, MA, USA) with an ESI ion source. Simultaneous MS1 and MS/MS (Full MS‐ddMS2 mode, data‐dependent MS/MS) acquisition was used. The parameters were as follows: sheath gas pressure, 40 arb; aux gas flow, 10 arb; spray voltage, 3.50 and −2.50 kV for ESI(+) and ESI(‐), respectively; capillary temperature, 325 °C; MS1 range, m/z 100–1000; MS1 resolving power, 70000 FWHM; number of data dependant scans per cycle, 3; MS/MS resolving power, 17500 FWHM; normalized collision energy, 30 eV; dynamic exclusion time, utomatic. The raw data were first converted to mzXML format by MSConvert in the ProteoWizard software package (v3.0.8789) and processed using R XCMS (v3.12.0) for feature detection, retention time correction, and alignment. Then, the data was corrected by the area normalization method to eliminate systematic errors. The metabolites were identified by accuracy mass and MS/MS data which were matched with HMDB (http://www.hmdb.ca), mass bank (http://www.massbank.jp/), KEGG (https://www.genome.jp/kegg/), LipidMaps (http://www.lipidmaps.org), mzcloud (https://www.mzcloud.org) and the metabolite database built by Panomix Biomedical Tech Co., Ltd. (Shuzhou, China). The molecular weight of metabolites was determined according to the m/z (mass‐to‐charge ratio) of parent ions in MS data. The molecular formula was predicted by ppm (parts per million) and adduct ion and then matched with the database to realize MS identification of metabolites. At the same time, the MS/MS data from the quantitative table of MS/MS data were matched with the fragment ions and other information of each metabolite in the database, to realize the MS/MS identification of metabolites. Two different multivariate statistical analysis models, unsupervised and supervised, were applied to discriminate the groups (PCA; PLS‐DA; OPLS‐DA) by R ropls (v1.22.0) package. The statistical significance of the *P* value was obtained by statistical tests between groups. Finally, combined with *P* value, VIP (OPLS‐DA variable projection importance), and FC (multiple of the difference between groups) to screen biomarker metabolites. By default, when *P* value < 0.05 and VIP value > 1, it was thought that metabolite was considered to have significant differential expression.

### Statistical Analysis

GraphPad Prism 8.0 was used for the statistical analysis. Data were expressed as mean ± SD or boxplots. A significant difference between the two groups was evaluated by the Mann–Whitney *U* test (non‐parametric) or two‐tailed unpaired Student's t‐test (parametric). For more than two groups of comparison, a one‐way analysis of variance (ANOVA) followed by the Tukey test was performed. ^*^ *p* < 0.05 indicates a significant difference. Other specific statistical analyses were mentioned in each methods section.

## Conflict of Interest

The authors declare no conflict of interest.

## Author Contributions

C.Z. and L.B. contributed equally to this work. C.Z., X.H., Y.F., N.Z., and C.Y. designed the study. C.Z. and L.B. performed all mouse animal experiments and all statistical analyses. R.S., Y.Z., K.W., S.S., H.L., Y.L., and K.C. assisted with animal experiments and experimental parameter determinations. Y.F., C.Z., C.Y., and X.H. obtained funding. C.Z. wrote the manuscript and all authors revised and approved the manuscript.

## Supporting information



Supporting Information

## Data Availability

The data that support the findings of this study are available from the corresponding author upon reasonable request.
