# Supplementary material for: Maternal Gut Inflammation Aggravates Acute Liver Failure Through Facilitating Ferroptosis via Altering Gut Microbial Metabolism in Offspring
Source: Adv Sci (Weinh). 2025 Jan 14;12(9):2411985. doi: 10.1002/advs.202411985 (PMC11884527; doi:10.1002/advs.202411985)
Supplement: Supplementary file 1 — Supporting Information [file ADVS-12-2411985-s001.docx]

**Supplemental information for**

**Maternal Gut Inflammation Aggravates Acute Liver Failure through Facilitating Ferroptosis via Altering Gut Microbial Metabolism in Offspring**

Caijun Zhao, Lijuan Bao, Ruping Shan, Yihong Zhao, Keyi Wu, Shan Shang, Haiqi Li, Yi Liu, Ke Chen, Naisheng Zhang, Cong Ye*, Xiaoyu Hu*, Yunhe Fu*

C. Zhao, K. Chen, C. Ye

Department of Gynecology

China-Japan Union Hospital of Jilin University

Changchun 130033, China

E-mail: [yecong0228@jlu.edu.cn](mailto:yecong0228@jlu.edu.cn)

C. Zhao, L. Bao, R. Shan, Y. Zhao, K. Wu, S. Shang, N. Zhang, X. Hu, Y. Fu

Department of Clinical Veterinary Medicine

College of Veterinary Medicine

Jilin University

Changchun 130062, China.

E-mail: [hxiaoyu@yeah.net](mailto:hxiaoyu@yeah.net); [fuyunhesky@sina.com](mailto:fuyunhesky@sina.com)

H. Li

Department of Neurology

China-Japan Union Hospital of Jilin University

Changchun 130033, China.

Y. Liu

Department of Orthopedic Center

The First Hospital of Jilin University

Changchun 130012, China.

**The files includes:**

**Supplementary figures S1-S7**

**Supplementary figures S1-S7**

**
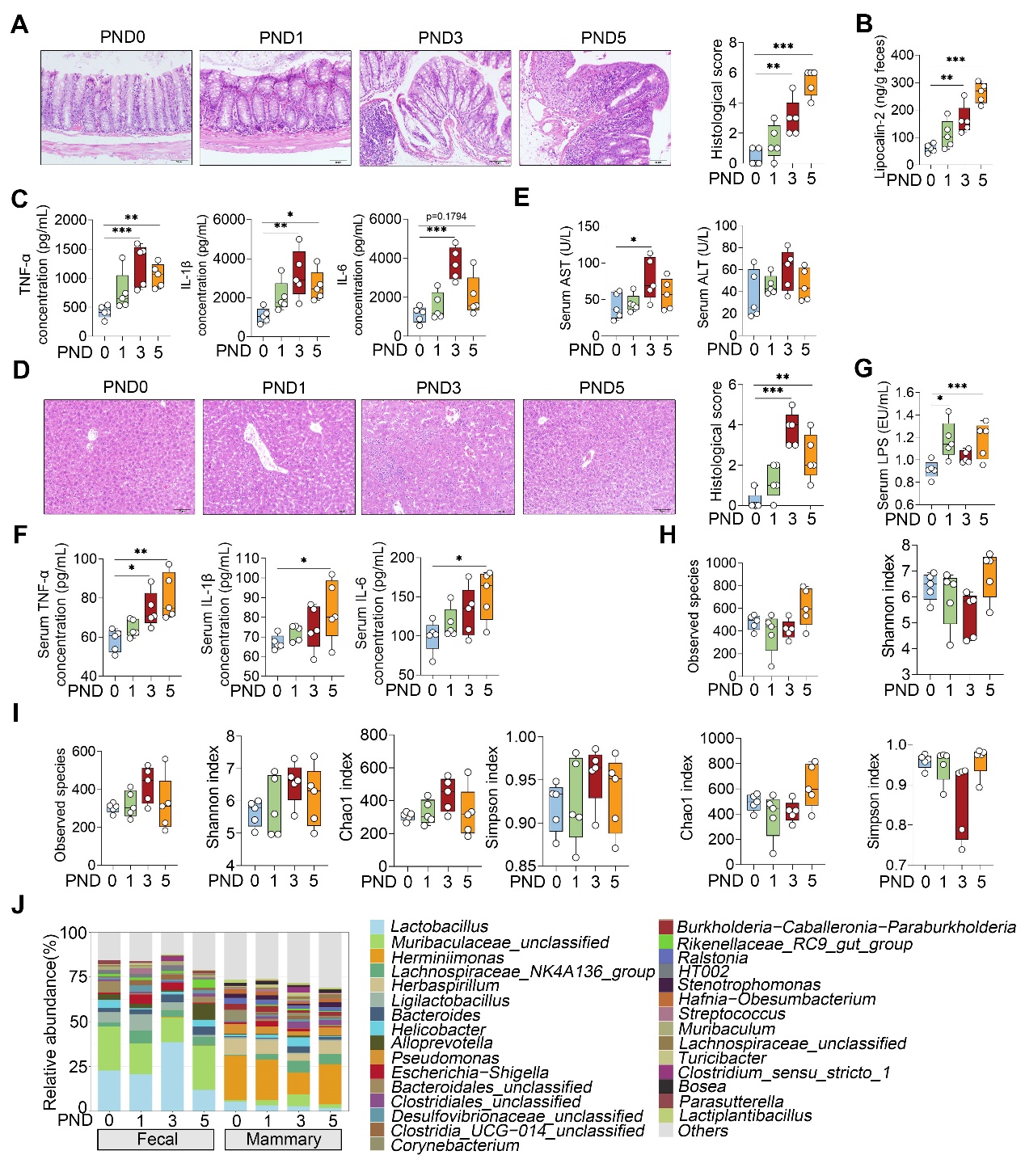
**

**Supplementary Figure. S1 Gut inflammation during lactation induces maternal systemic inflammation and changes the microbiota in maternal and offspring.** Lactating mice were treated with 3% DSS for three continuous days from PND 0 to PND 3, and the treated DSS was replaced with water alone from PND 3 to PND 5. (**A**) Representative H&E-stained images of colon and colonic histological score. (**B**) Fecal lipocalin-2 (LCN2) levels. (**C**) Proinflammatory cytokines of the mammary glands. (**D**) Representative H&E-stained images and histological score of the liver. (**E**) Serum ALT and AST levels. (**F** and **G**) Serum proinflammatory cytokine and LPS levels. (**H** and **I**) Microbial alpha diversity indices of gut (H) and mammary glands (I). (**J**) Fecal and mammary microbial compositions at the genus level. Data are expressed as boxplot (n=5-10). ***p* < 0.01 and ****p* < 0.001 by Kruskal-Wallis test followed by Dunn’s test (B, C and E-H) and Mann-Whitney *U* test (I). Scale bars, 50 μm.


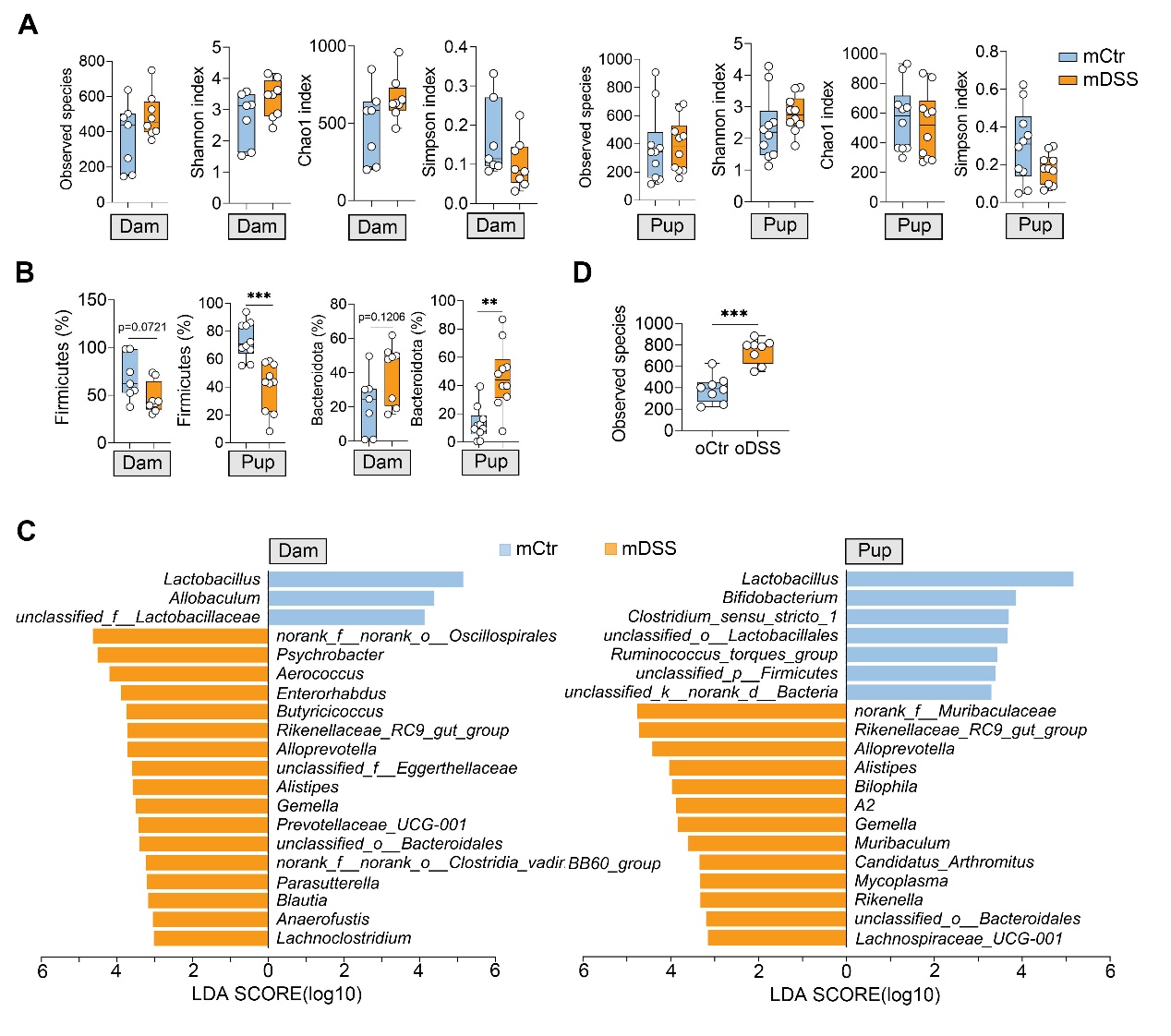


**Supplementary Figure. S2 MGI alters the gut microbiota of offspring. A**-**C.** Lactating mice were treated with 3% DSS for three continuous days from PND 0 to PND 3, followed by free access to drinking water until PND 21. (**A**) Alpha diversity indices of dams and pups at PND 21. (**B**) Relative abundances of *Fimicutes* and *Bacteroidota* in dams and pups. (**C**) Differential genera in different dams (left) and pups (right) were analyzed by LEfSe (LDA score (log 10) > 3). (**D**) Lactating mice were treated with 3% DSS for three continuous days from PND 0 to PND 3, followed by free access to drinking water until PND 21. The offspring were raised until PND 42 and observed species of the gut microbiota in adult offspring were detected. Data are expressed as boxplot (n=7-8). ****p* < 0.001 by Mann-Whitney *U* test (A, B and D).


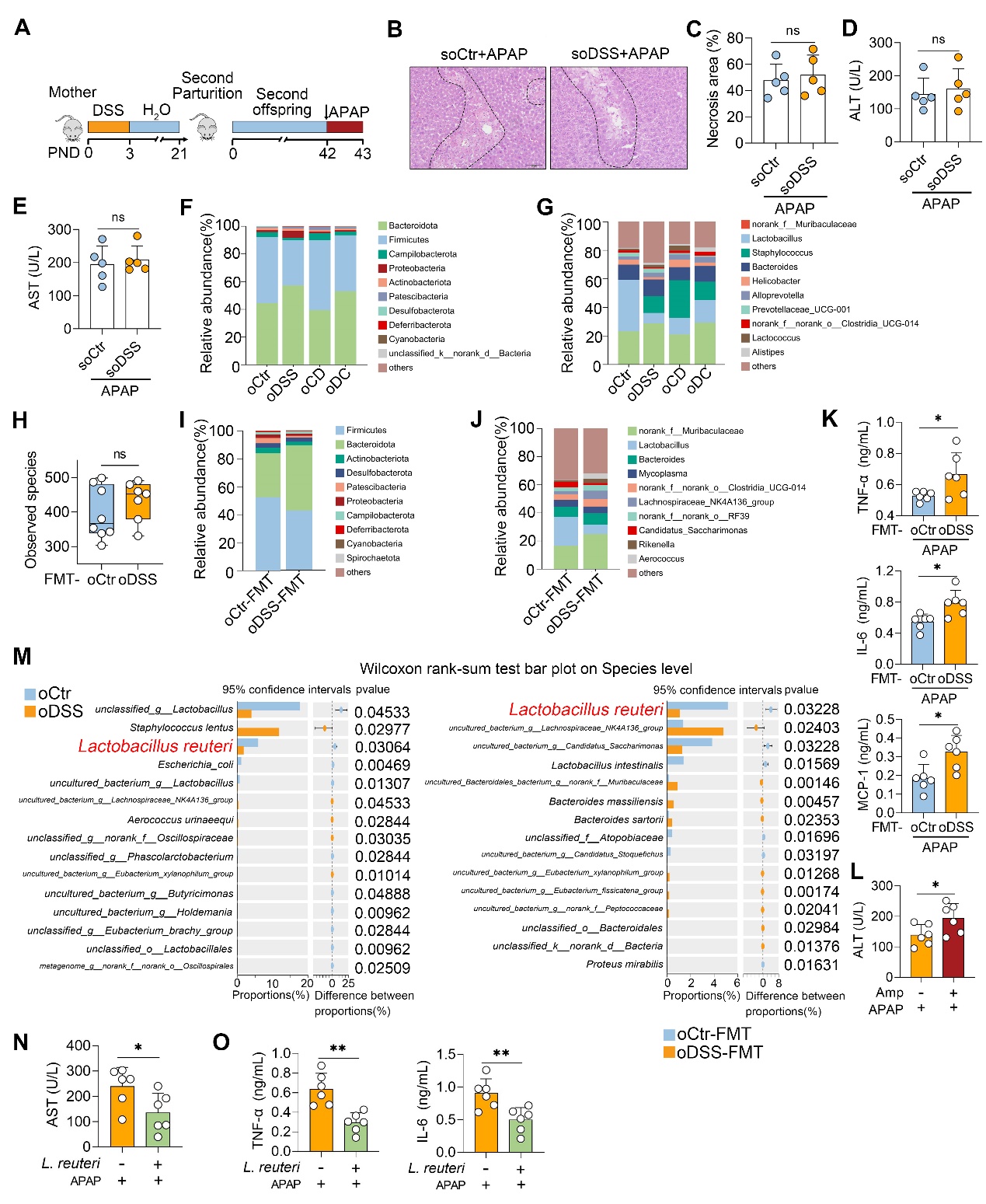


**Supplementary Figure. S3 MGI during lactation changes the gut microbiota, resulting in exacerbated ALF in offspring at adulthood.** (**A**) Schematic diagram of MGI on ALF in second offspring. Lactating mice were treated with 3% DSS for three continuous days to induce MGI, and these mice were mixed with male mice at PND 21 to produce second offspring. The second offspring were fed until adulthood and then treated with APAP (A-E). (**B** and **C**) Representative H&E-stained images and liver necrosis area analysis. (**D** and **E**) Serum ALT and ALT levels. (**F** and **G**) Three-week-old pups from control or DSS dams were placed in a single cage until PND 42, and gut microbial compositions at the phylum and genus levels were presented. **H**-**K**. Six-week-old mice were treated with ABX (200 mg/kg body weight of ampicillin, neomycin and metronidazole, 100 mg/kg vancomycin) for 5 days, followed by FMT for the next 21 days after replacing ABX with water for one day. At day 27, these mice were treated with 300 mg/kg APAP for 24 h. (**H**) Observed species of gut microbiota of FMT mice. (**I** and **J**) Gut microbial compositions at the phylum and genus levels of FMT mice. (**K**) Serum proinflammatory cytokines and ALT levels from indicated mice. (**L**) Conventional mice were treated with Amp (100 mg/kg) for five days, followed by APAP treatment and serum ALT level was detected. (**M**) Wilcoxon rank-sum test bar plot of 16S rRNA sequencing of fecal samples from donor and recipient mice in the oCtr and oDSS groups. (**N** and **O**) Serum AST and proinflammatory cytokine levels in *L. reuteri­*­-treated mice. The data are expressed as the means ± SDs (C-E, H, K, L and O, n=5-8). **p* < 0.05 and ***p* < 0.01 by two-tailed unpaired Student’s t test (C-E, H, K, L and O). ns, not significant. Scale bars, 50 μm.


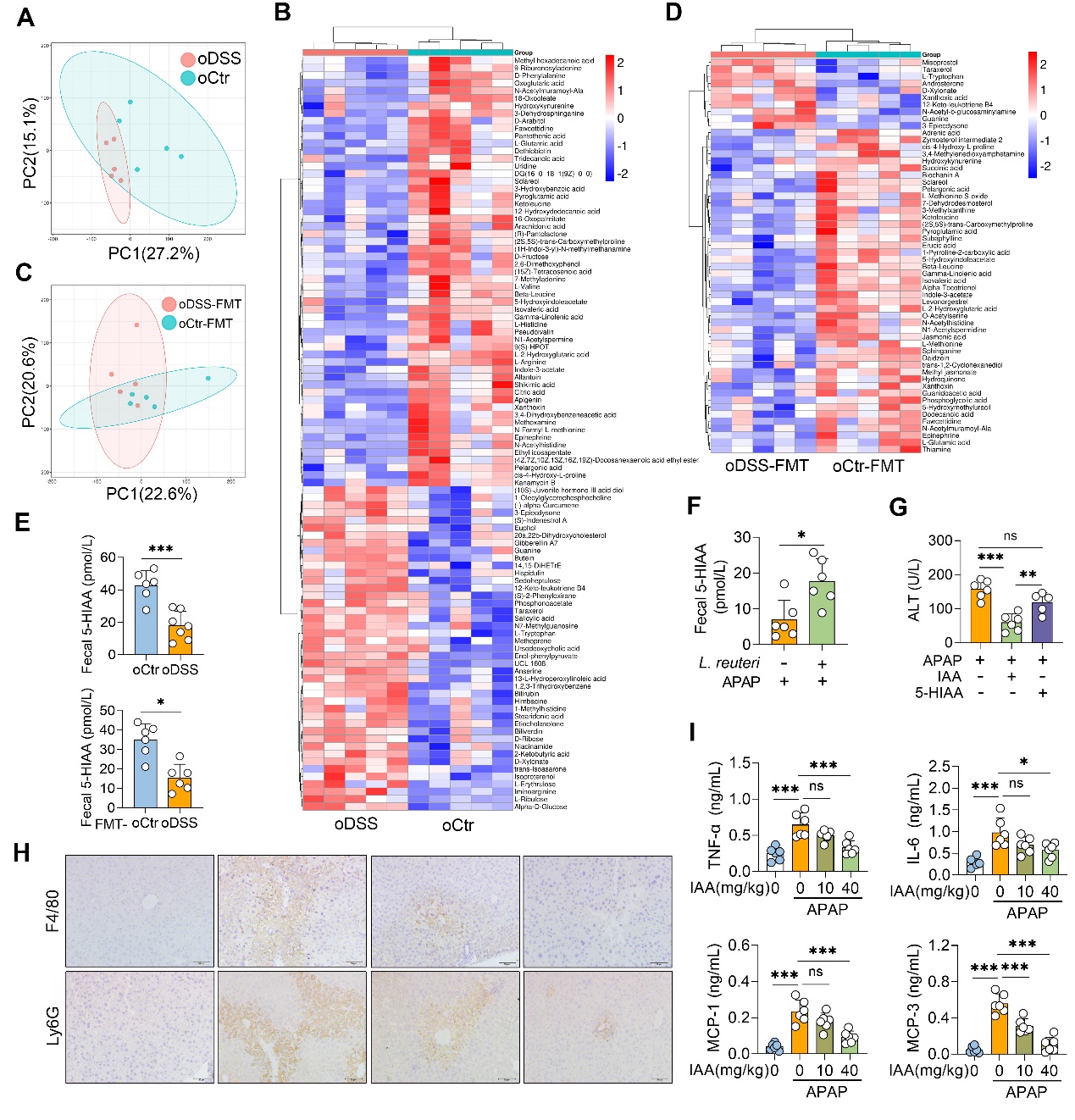


**Supplementary Figure. S4 MGI during lactation alters metabolic profiles of offspring.** (**A** and **C**) PCA score plots of metabolites in mice from donor and recipient mice in the oCtr and oDSS groups. (**B**) Heatmap of differential metabolites between the oCtr and oDSS groups. (**D**) Heatmap and count of differential metabolites between the oCtr-FMT and oDSS-FMT groups. (**E** and **F**) Fecal 5-HIAA levels of different offspring and FMT mice, as well as *L. reuteri*­-treated mice. (**G**) Serum ALT level of APAP-treated mice subjected to IAA and 5-HIAA treatments. **H-I.** Mice were orally treated with 10 mg/kg and 40 mg/kg IAA for 2 h, and then treated with 300 mg/kg APAP for 24 h. (**H**) Representative liver images of F4/80- and Ly6G-stained livers. (**I**) Serum proinflammatory cytokines levels, including TNF-α, IL-6, MCP-1, and MCP-3, from IAA-treated mice. The data are expressed as the means ± SDs (E-H, n=6). **p* < 0.05, ***p* < 0.01 and ****p* < 0.001 by two-tailed unpaired Student’s t test (E and F) and one-way ANOVA followed by Tukey’s test (G and I). ns, not significant.


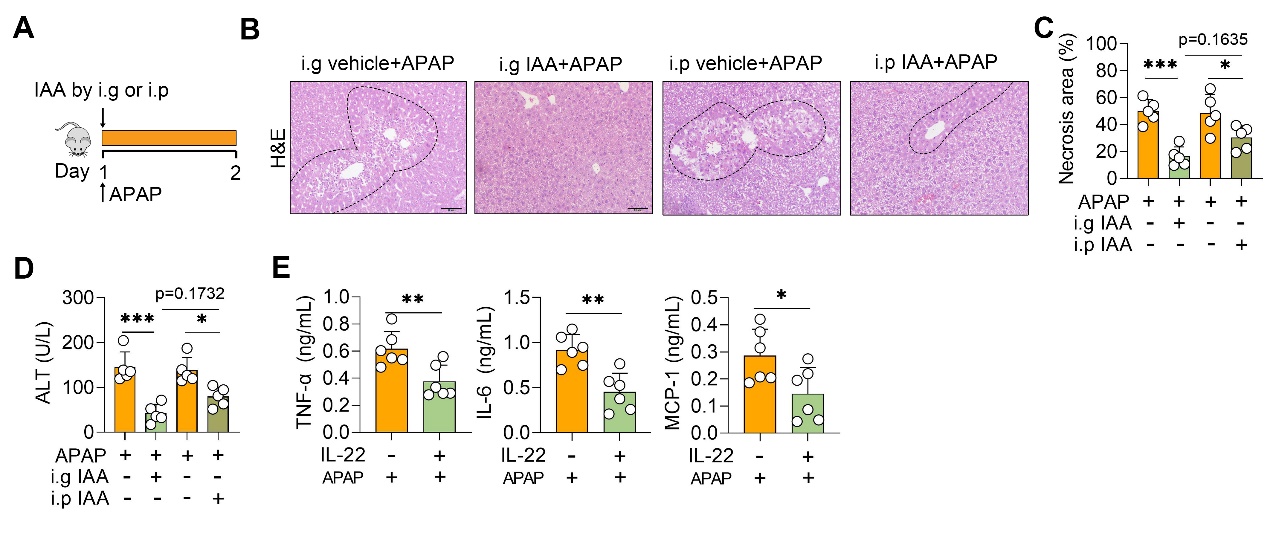


**Supplementary Figure. S5 IAA alleviates ALF by promoting IL-22 production.** (**A**) Schematic diagram of the effects of different IAA administration methods on ALF. Mice were pretreated with IAA (40 mg/kg) by oral gavage (i.g.) and intraperitoneal injection (i.p.) for 2 h, followed by APAP treatment for 24 h (A-D). (**B** and **C**) Representative H&E-stained images and liver necrosis area analysis. (**D**) Serum ALT level. (**E**) Serum proinflammatory cytokines levels of IL-22-treated mice. The data are expressed as the means ± SDs (n=5-6). **p* < 0.05, ***p* < 0.01 and ****p* < 0.001 by one-way ANOVA followed by Tukey’s test (C, D and E). Scale bars, 50 μm.


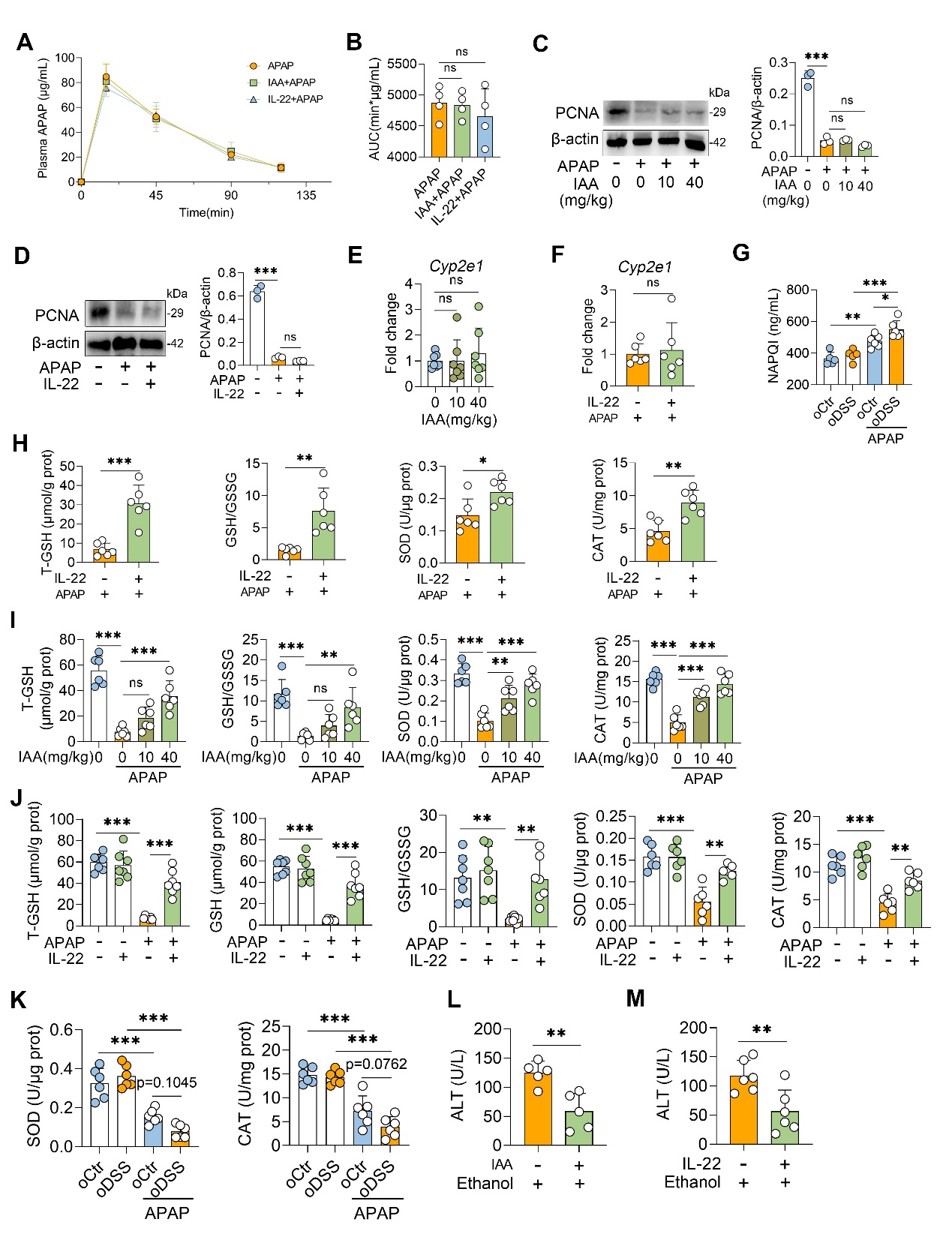


**Supplementary Figure. S6 IAA and IL-22 ameliorates APAP-induced oxidative stress.** (**A** and **B**) The mice were treated with IL-22 (1mg/kg) and IAA (40 mg/kg) for 2 h, followed by APAP (300 mg/kg) treatment and plasma APAP levels at 0, 15, 45, 90 and 120 min after APAP treatment and area under the curve (AUC) were detected. (**C** and **D**) Hepatic PCNA expressions of indicated mice. (**E** and **F**) Hepatic *Cyp2e1* expressions by qPCR of IAA- and IL-22-treated mice. (**G**) Different offspring were treated with APAP (300 mg/kg) for 1 h and hepatic NAPQI levels were detected. (**H**-**I**) Hepatic antioxidant levels including T-GSH, GSH, GSH/GSSG, SOD and CAT in indicated mice. (**J**) The AML-12 cells were treated with IL-22 (0.1μg/mL) for 2 h, followed by APAP (5mM) treatment for the next 24 h and hepatic antioxidant levels were detected. (K) Hepatic SOD and CAT levels in indicated offspring. (**L** and **M**) The mice were fasted for 12 h with free access to water, and then treated with IAA and IL-22 for 2 h, followed by 6 mg/kg ethanol treatment orally for 6 h, and serum ALT levels were detected. The data are expressed as the means ± SDs (n=3-7). **p* < 0.05, ***p* < 0.01 and ****p* < 0.001 by one-way ANOVA followed by Tukey’s test (B-E, G, I, J and K) and two-tailed unpaired Student’s t test (F, H, L and M). ns, not significant.


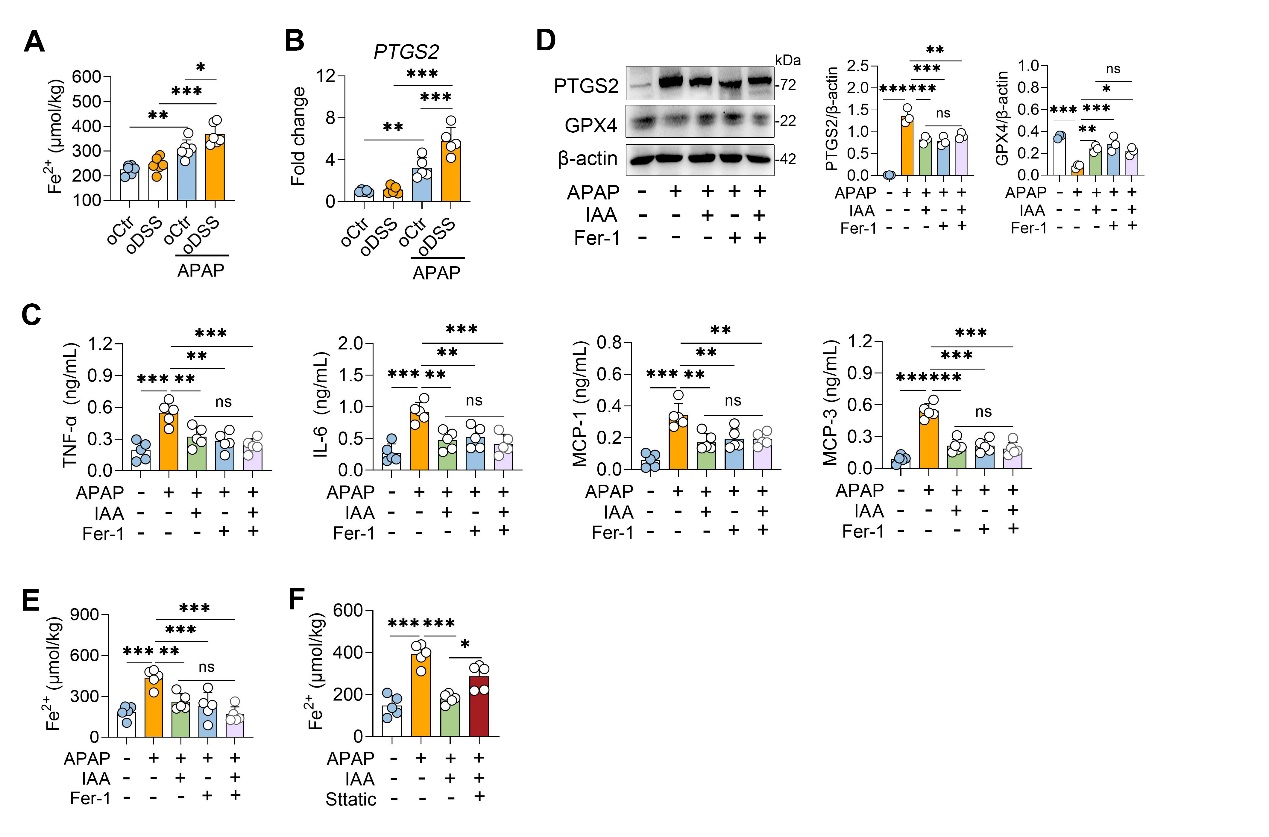


**Supplementary Figure. S7 IAA-mediated IL-22 production alleviates APAP-induced ferroptosis by activating STAT3.** (**A**) Hepatic Fe^2+^ levels of different offspring subjected to APAP treatment for 24 h. (**B**) Hepatic *PTGS2* levels of different offspring by qPCR. (**C**-**E**) The mice were treated with Fer-1 (10 mg/kg) with or without IAA (40 mg/kg) for 2 h, followed by APAP (300 mg/kg) treatment for 24 h and serum proinflammatory cytokine levels (C) and hepatic PTGS2 and GPX4 levels (D) were detected, as well as hepatic Fe^2+^ levels (E). (**F**) The mice were intraperitoneally injected with 10 mg/kg Stattic for 1 h prior to IAA (40 mg/kg) intervention, followed by APAP (300 mg/kg) treatment for 24 h and hepatic Fe^2+^ levels were detected. The data are expressed as the means ± SDs (n=3-5). **p* < 0.05, ***p* < 0.01 and ****p* < 0.001 by one-way ANOVA followed by Tukey’s test (A-F). ns, not significant.
